# Silicate minerals enhance the expression of genes related to mineral dissolution by *Priestia aryabhattai* strain C4-10

**DOI:** 10.1128/aem.02554-25

**Published:** 2026-01-26

**Authors:** Qi Sheng, Xin-Yi Zheng, Si-Han Yang, Wen Dong, Lin-Yan He, Xia-Fang Sheng

**Affiliations:** 1 College of Life Sciences, Nanjing Agricultural University 70578, Nanjing, China; Colorado School of Mines, Golden, Colorado, USA

**Keywords:** mineral-dissolving *Priestia aryabhattai*, dissolution of silicate minerals, transcriptomics, differentially expressed genes, mineral dissolution-related genes

## Abstract

Silicate mineral-microbe interactions are essential for soil formation, element biogeochemical cycles, and carbon sequestration. However, the molecular mechanisms by which gram-positive bacteria mediate mineral dissolution remain largely unexplored. Here, we characterized a highly effective mineral-dissolving *Priestia aryabhattai* strain, C4-10, for its biotite and lizardite dissolution activity, alongside the underlying molecular mechanisms. In the medium supplemented with biotite or lizardite, C4-10 significantly increased the Fe, Mg, and Si concentrations between 4 and 48 h of incubation compared to the controls. Notably, in the C4-10-inoculated medium supplemented with biotite or lizardite, significantly decreased pH values in the medium and increased cell counts and biofilm formation on the mineral surfaces were observed over 24 h of incubation. A comparative transcriptomic analysis indicated that significantly upregulated differentially expressed genes were enriched in pathways related to glyoxylate and dicarboxylate metabolism, amino acid biosynthesis, the tricarboxylic acid cycle, and ABC transporters in the presence of biotite. Additionally, the gene expression of *lutA_2* and *actP* associated with acid metabolism, *glgC* linked to biofilm formation, *gtaB_3* related to cell wall components, and *02676*, *levE*, and *glnQ* associated with transporters, was significantly upregulated in C4-10 in the presence of biotite or lizardite. Importantly, strong positive correlations were observed between the Fe or Mg concentrations and the relative expression levels of these genes during the biotite or lizardite dissolution process by C4-10. Our findings illustrate the involvement of multiple genes and metabolic pathways related to mineral dissolution, highlighting similar molecular mechanisms associated with both biotite and lizardite dissolution by C4-10.

**IMPORTANCE:**

To date, the molecular mechanisms underlying the dissolution of silicate minerals by gram-positive bacteria remain poorly understood. This study characterizes the mechanisms involved in biotite and lizardite dissolution by C4-10. C4-10 enhanced mineral dissolution through the production of organic acids, cell adsorption, and biofilm formation on mineral surfaces. The presence of biotite upregulated the expression of genes related to mineral dissolution and enriched metabolic pathways, including glyoxylate and dicarboxylate metabolism, amino acid biosynthesis, butanoate metabolism, the tricarboxylic acid cycle, and ABC transporters. Furthermore, significant correlations were observed between Fe or Mg concentrations in the medium and the expression levels of genes associated with acid metabolism, biofilm formation, cell wall metabolism, and transporters during the dissolution of biotite or lizardite by C4-10. Our results provide new insights into the interactions between silicate minerals and mineral-dissolving gram-positive bacteria, as well as the molecular mechanisms that facilitate in these processes.

## INTRODUCTION

The dissolution of silicate minerals, a common geological process on the Earth’s surface, is fundamental to soil formation and evolution, plant nutrition, biogeochemical cycling of elements, and carbon sequestration (1–4). The interactions between minerals and microorganisms have been pervasive since the emergence of life on Earth (5, 6). Microorganisms promote mineral dissolution by producing organic acids and metal-complexing ligands, altering redox conditions, and adhering to mineral surfaces (7–9). Numerous bacterial species have been documented to enhance the dissolution of silicate minerals (8, 10, 11). Bacterial exopolysaccharide can stimulate mineral dissolution, likely by forming metal-organic complexes on mineral surfaces and weakening metal-oxygen bonds within the mineral structure (12). The biofilm formation of *Pseudomonas aeruginosa* PA01 on the surface of potassium feldspar accelerates mineral dissolution (13). Biofilms of the rock-inhabiting fungus *Knufia petricola* strain A95 enhance olivine dissolution by preventing the formation of an amorphous layer (14). Moreover, microorganisms colonizing rocks form resilient biofilms known as biological rock crusts, which stabilize carbonate rock surfaces and protect weathered fronts (15). Bacterial adhesion to minerals promotes mineral dissolution by increasing proton concentrations, forming ion complexes at mineral surfaces, and catalyzing redox reactions (16, 17). Furthermore, the molecular mechanisms underlying bacteria-assisted mineral dissolution have been elucidated, revealing various genes and pathways implicated in this process (8, 18, 19).

*Bacillus* strains, including *B. megaterium*, *B. aryabhattai*, *B. flexus*, and *B. koreensis*, have been reclassified as *Priestia* strains (*P. megaterium*, *P. aryabhattai*, *P. flexus*, and *P. koreensis*) (20). These *Priestia* strains are widely distributed across various environments and are capable of dissolving silicate minerals (10, 21). For instance, *Priestia aryabhattai* SK1-7, isolated from the rhizosphere of *Populus alba* L., has been shown to enhance potassium feldspar dissolution by lowering pH and producing acids (22). However, the molecular mechanisms regulating silicate mineral dissolution in *Bacillus* strains remain poorly understood (8), hindering a comprehensive understanding of the interactions between silicate minerals and mineral-dissolving *Bacillus* strains. Uroz et al. (8) identified genes involved in the mineral dissolution of various bacterial strains, including *Burkholderia*, *Pseudomonas*, *Collimonas*, *Enterobacter*, *Paenibacillus*, *Rhizobium*, and *Serratia*. Notably, a novel type of glucose dehydrogenase linked to mineral dissolution was identified in *Collimonas pratensis* strain PMB3 (1) (18). However, limited information exists regarding mineral dissolution-related genes in *Priestia* strains. Yang et al. (22) reported significantly upregulated expressions of genes related to pyruvate metabolism, two-component system, DNA repair, and oxidative stress pathways in *P. aryabhattai* strain SK1-7 when exposed to potassium feldspar. Quantitative proteomic analysis revealed that *Bacillus subtilis* produces increased levels of oxidoreductases, facilitating serpentine transformation and promoting the synthesis of organic acids essential for mineral dissolution (23). Although specific genes and genetic systems involved in this process have been identified, few comprehensive studies have explored the general mechanisms employed by *Bacillus* strains for mineral dissolution. Furthermore, to date, no detailed studies have investigated the molecular mechanisms underlying the interactions between magnesium-bearing silicate minerals (such as biotite and lizardite) and the mineral-dissolving bacterium *P. aryabhattai* C4-10. In this study, we hypothesize that multiple genes and pathways are involved in the interactions between silicate minerals and strain C4-10, with distinct expression levels of these genes observed during the mineral dissolution process.

To date, our understanding of the molecular mechanisms involved in bacterial mineral dissolution is primarily based on studies of gram-negative bacteria (8). This study focuses on the isolation and characterization of a gram-positive, thermotolerant, and biotite-dissolving *P. aryabhattai* strain C4-10, evaluating its mineral-dissolving capabilities, mineral dissolution-related physiological activities, and genome characteristics. A comparative transcriptomic analysis was employed to identify differentially expressed genes (DEGs) in C4-10 when exposed to biotite, relative to gene expression in the absence of the mineral. The DEGs were subsequently annotated in the Gene Ontology (GO) database and KEGG pathways to assess their involvement in key metabolic pathways associated with biotite dissolution in C4-10. Furthermore, the expression levels of mineral dissolution-related genes during the dissolution processes of biotite and lizardite were measured, characterizing the biotite- or lizardite-induced expression of these genes. Collectively, our findings contribute valuable insights into the molecular mechanisms governing the interactions between silicate minerals and mineral-dissolving gram-positive bacterial strains.

## RESULTS

### Isolation of mineral-dissolving *Bacillus* strains

Ten gram-positive thermotolerant bacterial strains (C4-1–C4-10) were isolated from the rhizosphere soil of the camphor tree (*Cinnamomum camphora*) (Fig. S1). Inoculation with these strains significantly (*P* < 0.05) increased the Fe (1.03–17.85 μM) and Al (2.45–17.19 μM) concentrations in the biotite-supplemented medium, with enhancements ranging from 0.36- to 22.49-fold for Fe and 3.30- to 29.16-fold for Al, compared to the controls (Fig. S1). Additionally, inoculation with these strains significantly (*P* < 0.05) reduced the pH values in the medium (6.40–4.44) relative to the control (Fig. S1) Notably, strain C4-10 released the highest concentrations of Fe (17.85 μM) and Al (17.19 μM) from the mineral compared to the other strains (Fig. S1). Due to its superior ability to solubilize Fe and Al, strain C4-10 was selected for further investigation and identified as *P. aryabhattai* through 16S rRNA gene sequencing and whole-genome sequencing analyses (GenBank accession no. GCA_039631325.1).

### Silicate mineral dissolution and related physiological activity of C4-10

**Fig 1:**
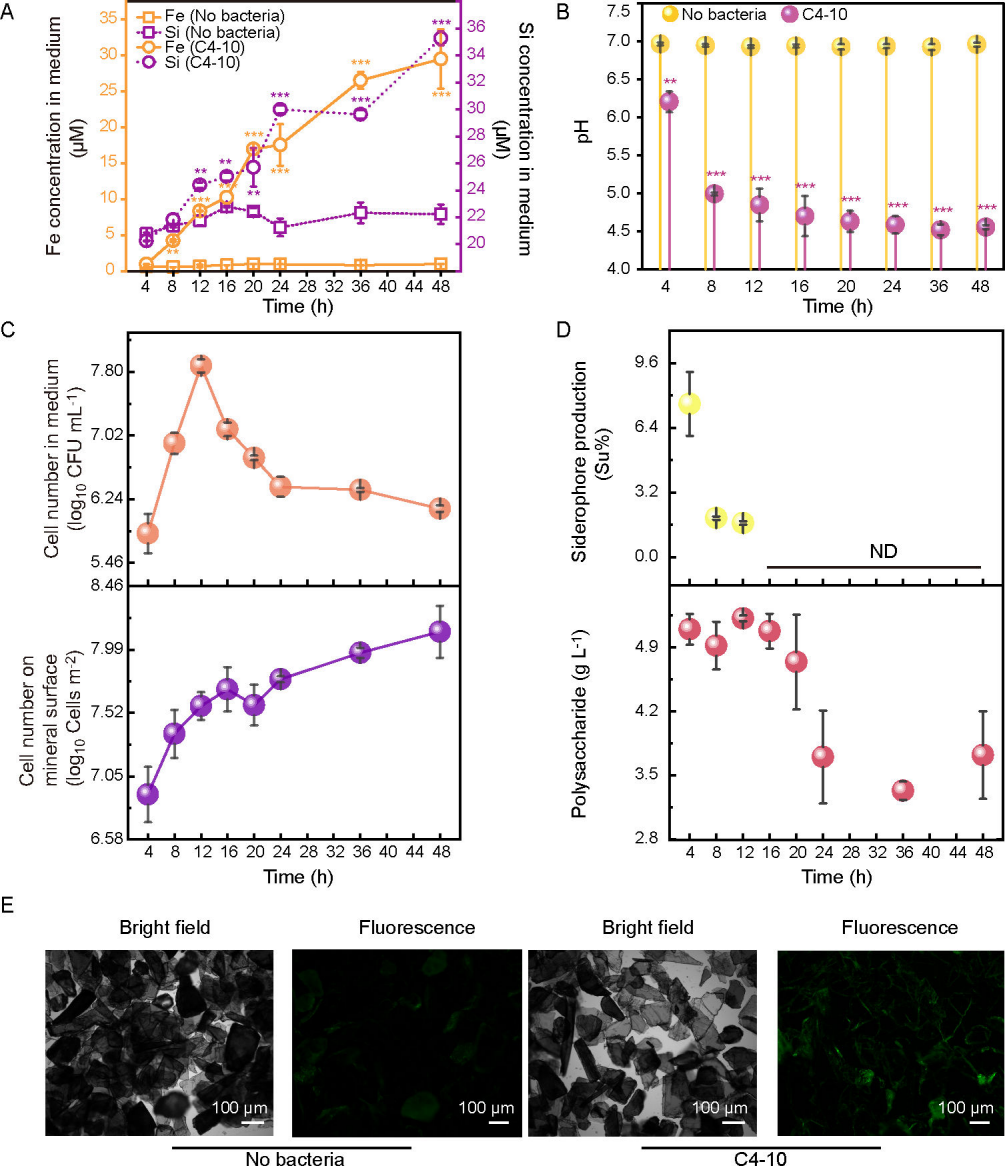
Changes in Fe and Si concentrations (**A**), pH (**B**), cell counts in the medium and on the biotite surface (**C**), siderophore and polysaccharide production (**D**) in the presence of strain C4-10 during 48 h of incubation, and the biofilm formation of strain C4-10 on the mineral surface were observed after 24 h of incubation under bright-field and fluorescence conditions (**E**). Error bars represent ±1 standard deviation (*n* = 3). **, *P* < 0.01; ***, *P* < 0.001. ND indicates no detection of siderophore production in the medium.

In the medium supplemented with biotite, inoculation with C4-10 significantly increased Fe concentrations (0.99*–*29.5 μM) by 0.62- to 30-fold between 4 and 48 h and Si concentrations (24.4–35.3 μM) by 0.12- to 0.58-fold between 12 and 48 h compared to the controls (Fig. 1A). The pH values in C4-10-inoculated medium decreased from 6.21 to 4.52 over the same period (Fig. 1B). The cell counts of C4-10 in the medium increased from 5.82 to 7.88 log_10_ CFU mL^−1^ between 4 and 12 h, followed by a gradual decline between 16 and 48 h, whereas the cell counts of C4-10 on the biotite surface increased from 6.91 to 8.12 log_10_ cells m^−2^ between 4 and 48 h (Fig. 1C). The cell-associated Fe contents significantly (*P* < 0.01) increased from 0.04 to 0.08 μg mg^−1^ between 12 and 24 h (Fig. S2). The Fe and Si concentrations, as well as pH values in the controls, did not exhibit significant (*P* > 0.05) changes (Fig. 1A and B). The siderophore levels in the medium ranged from 7.6% to 1.7% between 4 and 12 h but were not detected between 16 and 48 h (Fig. 1D). The exopolysaccharide (EPS) concentrations in the C4-10-inoculated medium varied from 3.52 to 5.15 g L^−1^ during the incubation period of 4−48 h (Fig. 1D). Biofilms on the surface of biotite inoculated with C4-10 were also observed after 24 h of incubation (Fig. 1E). Furthermore, the cell counts of C4-10 in the medium devoid of biotite ranged from 5.62 to 7.38 log_10_ CFU mL^−1^ between 4 and 24 h (Fig. S3A). In the C4-10-inoculated medium, malic acid concentrations (245−598 μM) significantly (*P* < 0.05) increased by 0.68- to 0.86-fold in the presence of biotite compared to its absence between 4 and 8 h (Fig. S3B). The concentrations of acetic (123−250 μM) and oxalic (327−649 μM) acids also significantly (*P* < 0.05) increased by 0.48- to 0.70-fold and 0.23- to 0.85-fold, respectively, in the C4-10-inoculated medium containing biotite compared to the absence of the mineral between 12 and 24 h (Fig. S3C and D).

**Fig 2:**
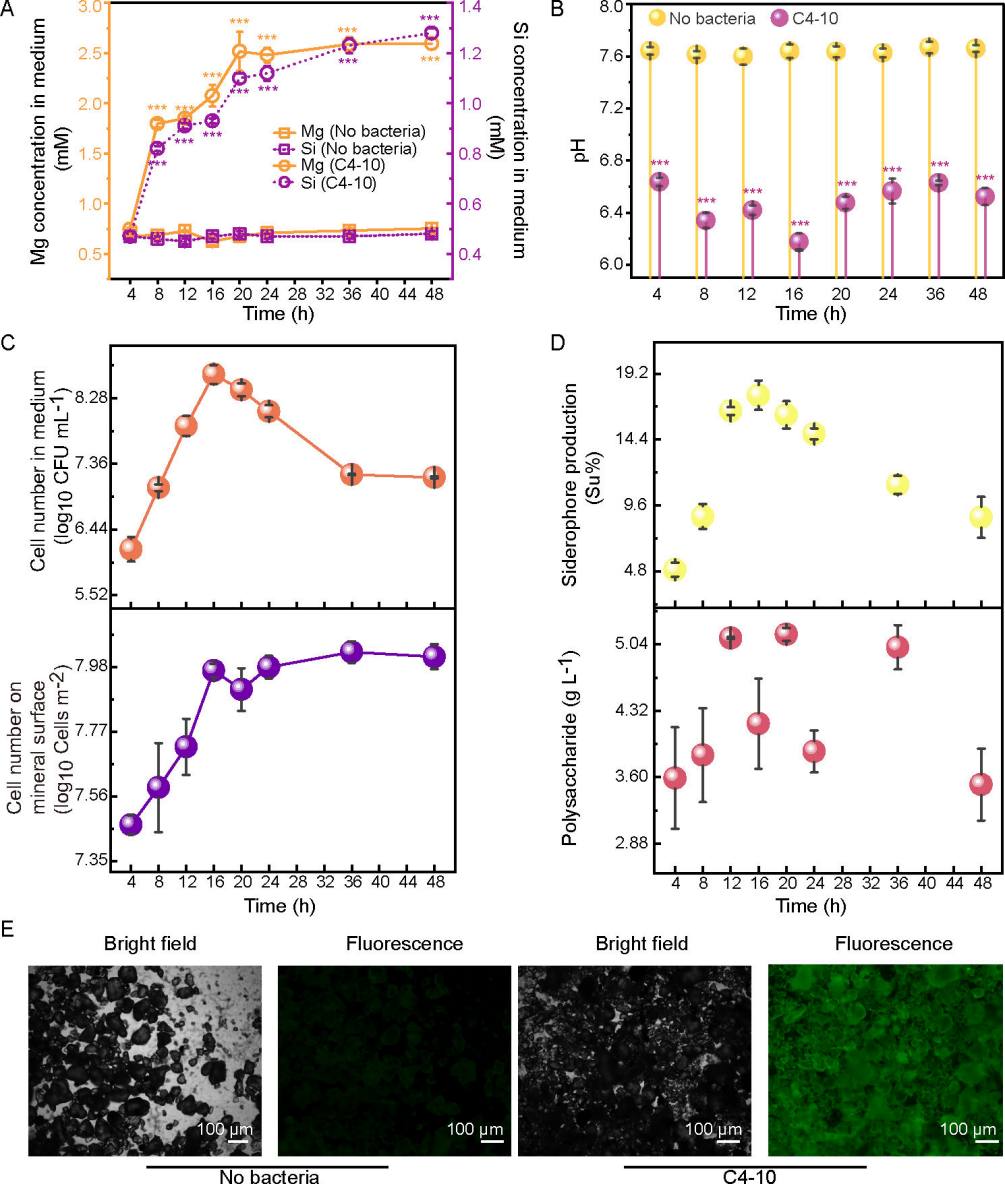
Changes in Mg and Si concentrations (**A**), pH (**B**), cell counts in the medium and on the lizardite surface (**C**), siderophore and polysaccharide production (**D**) in the presence of strain C4-10 during 48 h of incubation, and the biofilm formation of strain C4-10 on the mineral surface were observed after 24 h of incubation under bright-field and fluorescence conditions (**E**). Error bars represent ±1 standard deviation (*n* = 3). ***, *P* < 0.001.

To gain further insight into the effect of C4-10 on silicate mineral dissolution and the underlying mechanisms, the dissolution of lizardite and related physiological activities of C4-10 were also investigated in this study. In the medium supplemented with lizardite, inoculation with C4-10 significantly increased Mg concentrations (1.80−2.60 mM) by 1.60- to 2.50-fold and Si concentrations (0.82−1.27 mM) by 0.78- to 1.65-fold between 8 and 48 h compared to the controls (Fig. 2A). The pH values in the C4-10-inoculated medium decreased over time between 4 and 48 h (Fig. 2B). The cell counts of C4-10 increased from 6.16 to 8.62 log_10_ CFU mL^−1^ between 4 and 16 h and decreased from 8.40 to 7.17 log_10_ CFU mL^−1^ between 20 and 48 h (Fig. 2C). The cell counts of C4-10 on the lizardite surfaces increased from 7.47 to 7.97 log_10_ cells m^−2^ between 4 and 16 h and remained stable (7.91−8.03 log_10_ cells m^−2^) between 20 and 48 h (Fig. 2C). The cell-associated Mg contents significantly (*P* < 0.01) increased from 0.61 to 2.22 μg mg^−1^ between 12 and 24 h (Fig. S2). The Mg and Si concentrations, as well as pH values in the controls, did not exhibit significant (*P* > 0.05) changes (Fig. 1A and B). The siderophore levels in the medium significantly (*P* < 0.05) increased over time (4.9%–17.6%) between 4 and 16 h, then gradually decreased (16.2%–8.7%) between 20 and 48 h (Fig. 2D). The EPS concentrations in C4-10-inoculated medium ranged from 3.52 to 5.15 g L^−1^ between 4 and 48 h of incubation (Fig. 2D). Moreover, biofilms on the lizardite surface with the inoculation of C4-10 were observed after 24 h of incubation (Fig. 2E). These findings demonstrate that C4-10 promotes the dissolution of biotite and lizardite through the production of acids, siderophores, EPS, and cell adsorption on the mineral surfaces.

### Genome characteristics of C4-10

A comprehensive analysis of the whole-genome sequence of C4-10 was conducted to investigate its potential mechanisms related to mineral dissolution. The genetic map of C4-10 is presented in Fig. S4. Gene prediction for C4-10 was performed using Glimmer (version 3.02) (http://ccb.jhu.edu/software/glimmer/index.shtml), which identified 5,672 genes, with an average gene length of 809 bp. Within the C4-10 genome, 27, 28, 44, 44, and 44 genes associated with 2-oxocarboxylic acid metabolism, the tricarboxylic acid (TCA) cycle, glyoxylate and dicarboxylate metabolism, butanoate metabolism, and the pentose phosphate pathway were identified, respectively. These genes may contribute to the organic acid metabolism of the strain. Furthermore, there were 48, 32, and 41 genes related to fatty acid biosynthesis, peptidoglycan biosynthesis, and amino sugar and nucleotide sugar metabolism, respectively, in the C4-10 genome, potentially responsible for the cell wall/membrane components of the strain. In addition, the genome of strain C4-10 contained 1, 15, and 112 genes related to the biosynthesis of siderophore group non-ribosomal peptides, biofilm formation, and ABC transporters, respectively. Genes and metabolic pathways associated with acid and siderophore production, biofilm formation, cell wall/membrane biogenesis, and transporters in the genome of C4-10 likely play significant roles in mineral dissolution activity of the strain.

### Transcriptomic analysis reveals DEGs in C4-10

**Fig 3:**
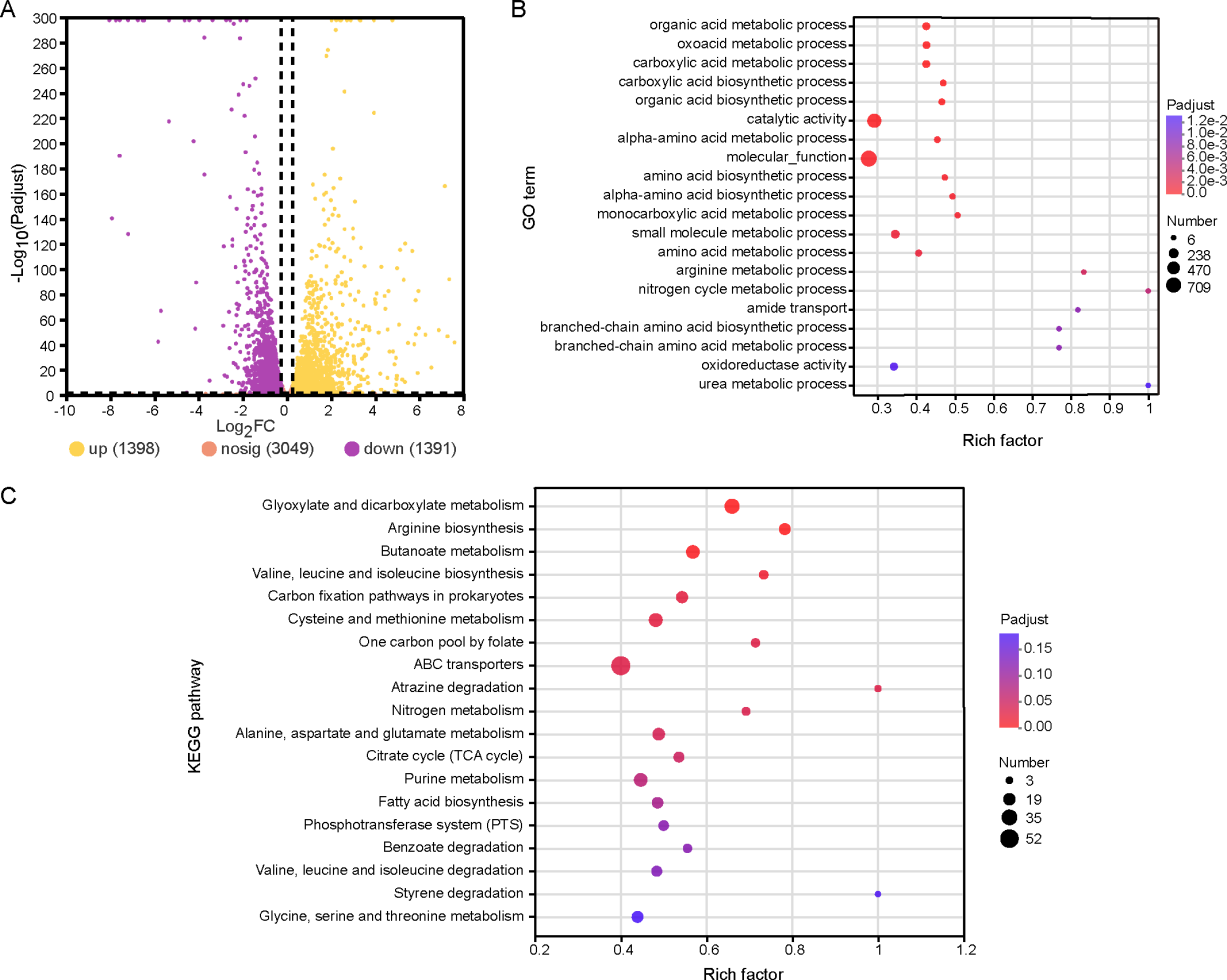
Transcriptomic analysis of C4-10 cultivated in BHm in the presence of biotite compared with that in the absence of the mineral. Upset Venn diagram showing DEGs, with upregulated and downregulated expression (**A**). GO (**B**) and KEGG (**C**) enrichment analysis of regulated DEGs.

To further investigate the changes in gene expression of C4-10 during biotite dissolution and the associated molecular mechanisms, DEGs exhibiting a fold change greater than 1.2 and a corrected *P* value below 0.05 were analyzed based on the comparative transcriptomes of the strain cultivated in BHm with biotite versus those cultivated in its absence. After 8 h of incubation, a comparison between C4-10 cells grown with and without biotite identified 1,398 upregulated and 1,391 downregulated genes in the presence of biotite (Fig. 3A). The upregulated genes were predominantly involved in amino acid transport and metabolism, carbohydrate transport and metabolism, energy production and conversion, two-component systems, and lipid transport and metabolism. Conversely, the downregulated genes were mainly linked to translation, ribosomal structure and biogenesis, transcription, coenzyme transport and metabolism, and inorganic ion transport and metabolism. This study primarily focused on the upregulated genes to elucidate the molecular mechanisms underlying biotite dissolution by C4-10. GO analysis indicated that the categories with the highest numbers of DEGs included catalytic activity, binding, metabolic process, and cellular process (Fig. S5A). Furthermore, KEGG analysis revealed that the categories with the highest numbers of DEGs encompassed carbohydrate metabolism, amino acid metabolism, membrane transport, energy metabolism, and signal transduction (Fig. S5B).

### GO and KEGG enrichment analysis of DEGs

GO enrichment analysis indicated that the upregulated DEGs were significantly (*P* < 0.001) enriched in the biosynthetic processes of organic acids, carboxylic acids, and amino acids, as well as catalytic activity and molecular function (Fig. 3B). In addition, KEGG enrichment analysis demonstrated that the upregulated DEGs were significantly (*P* < 0.05) enriched in various metabolic pathways, including glyoxylate and dicarboxylate metabolism, biosynthesis of amino acids (arginine, valine, leucine, and isoleucine), butanoate metabolism, the TCA cycle, and ABC transporters (Fig. 3C). These findings imply a complex cellular response involving multiple genes and metabolic pathways in the biotite dissolution process by C4-10.

### DEGs involved in key and related metabolic pathways related to biotite dissolution by C4-10

DEGs associated with critical metabolic pathways related to biotite dissolution in C4-10 are summarized in Table 1. Enrichment analysis revealed that metabolic pathways linked to acid metabolism (7 DEGs), biofilm formation (2 DEGs), cell wall/membrane metabolism (6 DEGs), and transporters (12 DEGs) in C4-10 exhibited increased gene expression levels in the presence of biotite. This suggests that multiple genes and metabolic pathways are involved in mineral dissolution by C4-10. Subsequently, seven upregulated DEGs (*lutA_2*, *actP*, *glgC*, *gtaB_3*, *02676*, *levE*, and *glnQ*) were selected for further analysis based on their associations with various mineral dissolution-related pathways (Table 1). These genes are implicated in several processes: transformation of glycolate to glyoxylate (potentially linked to the TCA cycle and organic acid synthesis), acetate secretion, polysaccharide biosynthesis, UDP-linked peptidoglycan assembly, gluconate transmembrane transport, transformation of fructose to 1-phosphate-fructose, and transport of aspartic acid, glutamic acid, and glutamine. In the context of biotite presence, these genes play roles in acid metabolism, biofilm formation, cell wall/membrane metabolism, and transport in the context of biotite presence (Table 1). To elucidate the molecular mechanisms underlying silicate mineral dissolution by C4-10, the expression levels of these seven target genes of C4-10 were assessed in the presence of biotite or lizardite.

**TABLE 1:** Genes upregulated in C4-10 in the presence of biotite grouped by metabolic pathways based on KEGG pathway enrichment analysis^*b*^

Pathway	Gene name	Gene ID	Function or description of the product	Fold change^*a*^
Acid metabolism				
	*citA*	Gene03098	Citrate synthase/methylcitrate synthase	169***
	*pckA*	Gene04826	Phosphoenolpyruvate carboxykinase (ATP)	46.49***
	*glcB*	Gene02911	Malate synthase G	11.98***
	*lutA_2*	Gene02671	Glycolate dehydrogenase iron-sulfur subunit	9.96***
	*acsA_2*	Gene04776	Acetate-CoA ligase	6.14***
	*actP*	Gene00930	Cation acetate symporter	3.73***
	*pycA*	Gene01365	Pyruvate carboxylase	2.30***
Biofilm formation				
	*glgC*	Gene04861	Glucose-1-phosphate adenylyltransferase	2.38***
	*glgD*	Gene04860	Sugar phosphate nucleotidyltransferase	2.13***
Cell wall/membrane metabolism				
	–	Gene02398	Cell wall hydrolase	11.48*
	*yocH_2*	Gene03010	LysM peptidoglycan-binding domain-containing protein	7.92***
	*capA_2*	Gene02858	Capsule biosynthesis protein	5.12***
	*gtaB_3*	Gene01501	UTP-glucose-1-phosphate uridylyltransferase GalU	4.88***
	*yohK*	Gene01301	LrgB family protein	3.92***
	*dacF*	Gene04359	D-alanyl-D-alanine carboxypeptidase	3.07***
Transporter				
	–	Gene02676	GntP family permease	50.9***
	*levE*	Gene01576	Mannose/fructose/N-acetylgalactosamine-transporter subunit IIB	46.18***
	*levD*	Gene01575	Mannose/fructose/sorbose PTS transporter subunit IIA	42.34***
	*sorA*	Gene01577	PTS mannose/fructose/sorbose transporter subunit IIC	35.27***
	*lacF_1*	Gene00951	Sugar ABC transporter permease	10.60**
	*glnQ*	Gene00320	Amino acid ABC transporter ATP-binding protein	5.30***
	*msmE_2*	Gene01440	Extracellular solute-binding protein	4.03***
	*glnM*	Gene00322	Amino acid ABC transporter permease	3.59***
	*idnT_1*	Gene00779	2-keto-3-deoxygluconate permease	3.44***
	*melE*	Gene02808	Extracellular solute-binding protein	3.07**
	*msmX_2*	Gene01441	Sn-glycerol-3-phosphate ABC transporter ATP-binding protein	3.01*
	*ngcF*	Gene01439	Sugar ABC transporter permease	2.51***

^*a*^ *P < 0.05, **P < 0.01, ***P < 0.001.

^*b*^ –, no gene name.

### Mineral-induced target gene expression analyzed by RT-qPCR

**Fig 4:**
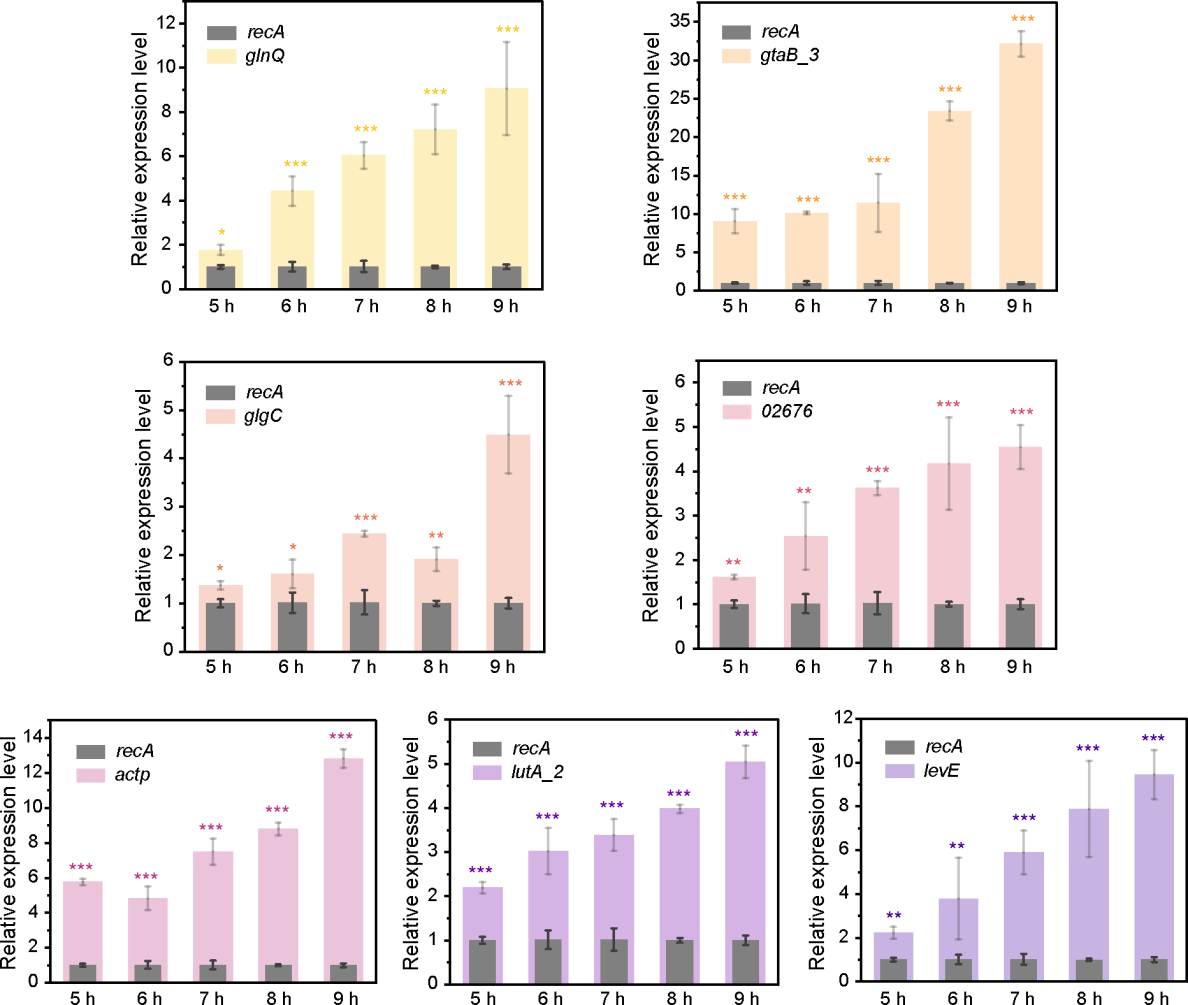
Relative expression of mineral dissolution-related genes (*glnQ*, *gtaB_3*, *glgC*, *02676*, *actP*, *lutA_2*, and *levE*) in C4-10 during the biotite dissolution process, analyzed by RT-qPCR at 5, 6, 7, 8, and 9 h of incubation. Error bars represent ±1 standard deviation (*n* = 3). *, *P* < 0.05; **, *P* < 0.01; ***, *P* < 0.001.

**Fig 5:**
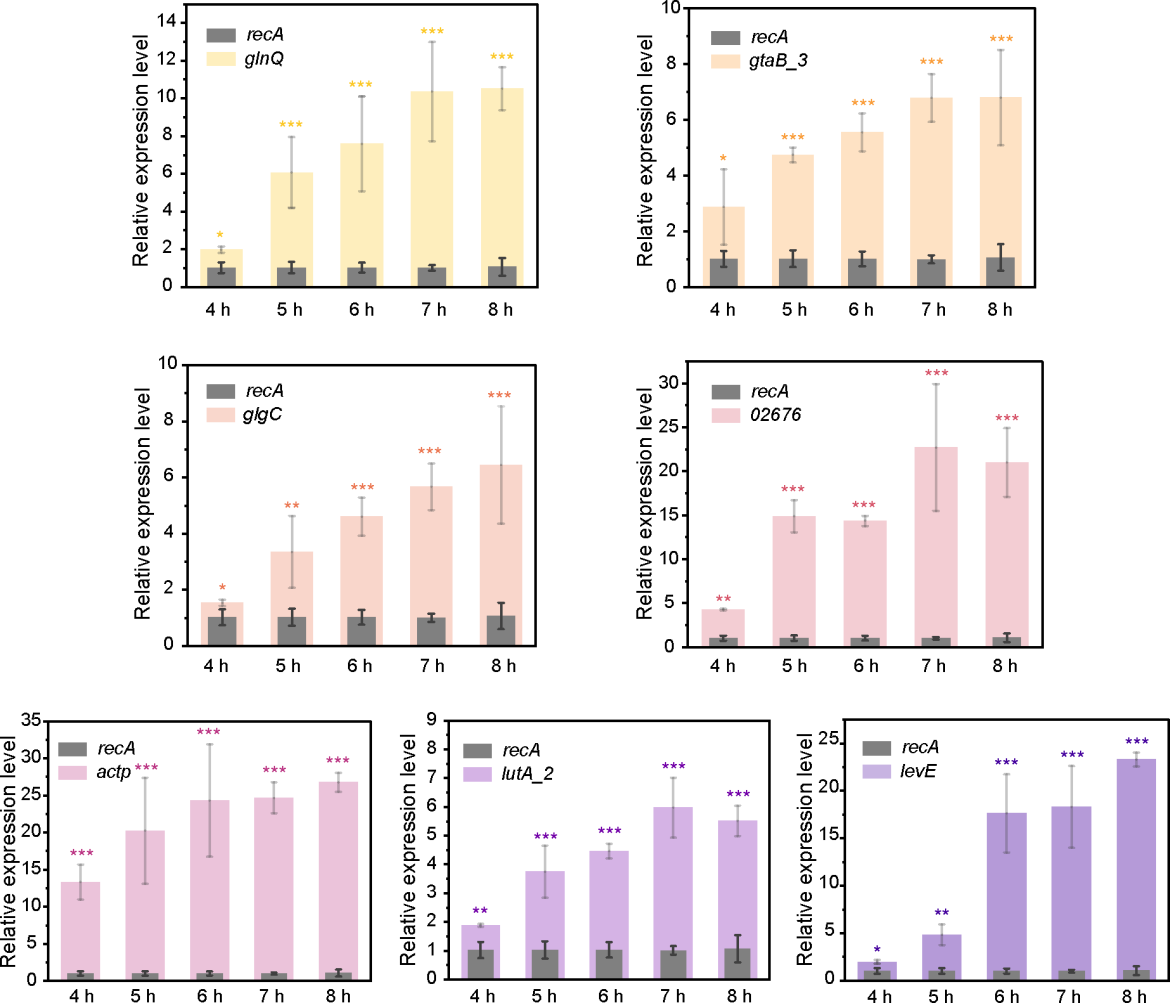
Relative expression of mineral dissolution-related genes (*glnQ*, *gtaB_3*, *glgC*, *02676*, *actP*, *lutA_2*, and *levE*) in C4-10 during the lizardite dissolution process, analyzed by RT-qPCR at 4, 5, 6, 7, and 8 h of incubation. Error bars represent ±1 standard deviation (*n* = 3). *, *P* < 0.05; **, *P* < 0.01; ***, *P* < 0.001.

To evaluate the accuracy of the DEGs identified from the transcriptomic data, seven selected DEGs, including *lutA_2* (encoding glycolate dehydrogenase iron-sulfur subunit), *actP* (encoding cation acetate symporter), *glgC* (encoding glucose-1-phosphate adenylyltransferase), *gtaB_3* (encoding UTP-glucose-1-phosphate uridylyltransferase), *02676* (encoding GntP family permease), *levE* (encoding mannose/fructose/N-acetylgalactosamine-transporter subunit), and *glnQ* (encoding amino acid ABC transporter ATP-binding protein) in C4-10, were analyzed using RT-qPCR in the presence of biotite or lizardite. During the biotite dissolution process by C4-10, the relative expression levels of the aforementioned DEGs increased significantly, ranging from 1.2- to 4.1-fold for *lutA_2*, 3.7- to 11.8-fold for *actP*, 0.4- to 3.5-fold for *glgC*, 8.1- to 31.1-fold for *gtaB_3*, 0.6- to 3.6-fold for *02676*, 1.2- to 8.5-fold for *levE*, and 0.8- to 8.1-fold for *glnQ*, respectively, between 5 and 9 h, coinciding with the acceleration of Fe release from biotite by C4-10 (Fig. 4). Similarly, during the dissolution process of lizardite by C4-10, the relative expression levels of the DEGs also exhibited significant increases: *lutA_2* showed a fold change of 0.8–4.9, *actP* 12.1–24.0, *glgC* 0.5–5.0, *gtaB_3* 3 1.8–5.7, *02676* 3.2–21.5, *levE* 1.0–20.8, and *glnQ* 0.9–8.8, between 4 and 8 h, as C4-10 rapidly released Mg from lizardite (Fig. 5). These RT-qPCR results corroborated the transcriptomic data, indicating positive induction of these genes by biotite or lizardite. These results further suggest that genes involved in acid metabolism, biofilm formation, cell wall metabolism, and transport may contribute to the dissolution process of both biotite and lizardite by C4-10.

### Mineral-induced target gene expression in C4-10 correlates with biotite and lizardite solubilization

**Fig 6:**
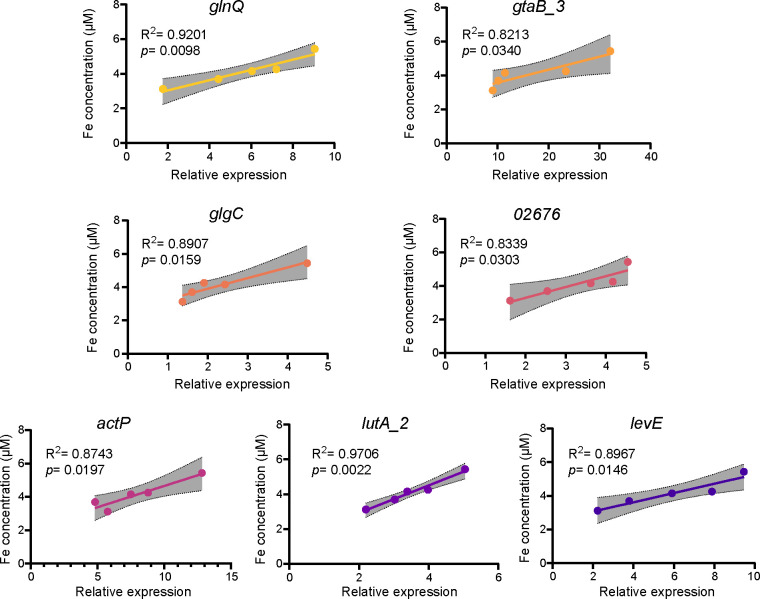
Correlation analyses of Fe concentrations in the medium and expression levels of *glnQ*, *gtaB_3*, *glgC*, *02676*, *actP*, *lutA_2*, and *levE* in C4-10 during the biotite dissolution process. Error bars represent ±1 standard deviation (*n* = 3).

**Fig 7:**
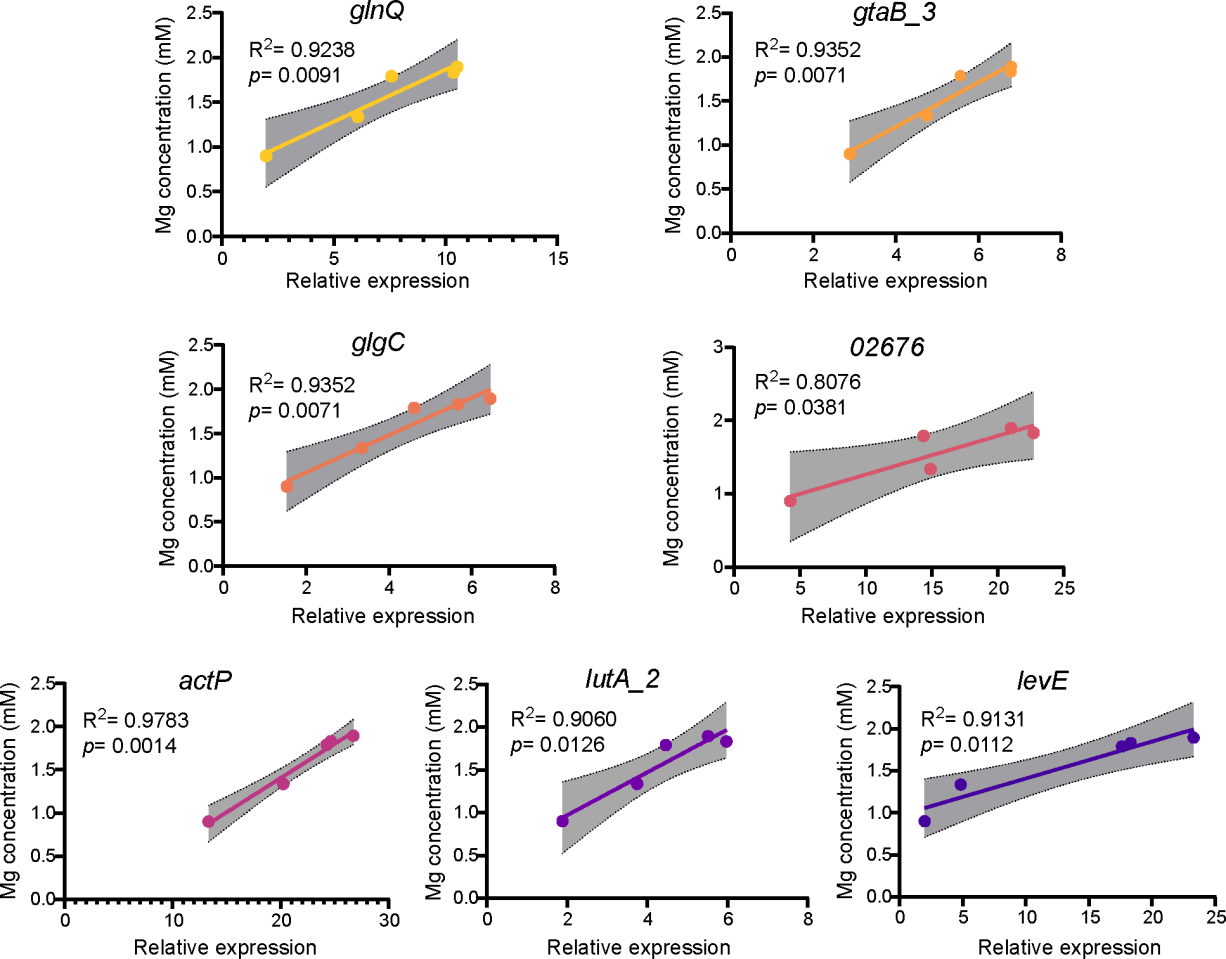
Correlation analyses of Mg concentrations in the medium and expression levels of *glnQ*, *gtaB_3*, *glgC*, *02676*, *actP*, *lutA_2*, and *levE* in C4-10 during the lizardite dissolution process. Error bars represent ±1 standard deviation (*n* = 3).

To further investigate the roles of the seven DEGs (*lutA_2*, *actP*, *glgC*, *gtaB_3*, *02676*, *levE*, and *glnQ*) in the dissolution of biotite and lizardite by C4-10, we evaluated the correlations between Fe or Mg concentrations and relative expression levels of these genes throughout the mineral dissolution process (Fig. S6 and S7). The inoculation with C4-10 significantly (*P* < 0.05) increased Fe concentrations (3.12–5.43 μM) in the presence of biotite between 5 and 9 h of incubation, and Mg concentrations (1.34–1.90 mM) in the presence of lizardite between 4 and 8 h (Fig. S6A and S7A). Notably, significant positive correlations were identified between Fe concentrations and the relative expression levels of these seven genes (*R*^2^ = 0.8213–0.9706, *P* = 0.0022–0.0340) and between Mg concentrations and the relative expression levels of these genes (*R*^2^ = 0.807–0.978, *P* = 0.001–0.038) (Fig. 6 and 7). Collectively, these findings underscore the substantial role of these genes in the dissolution process of biotite and lizardite by C4-10.

### The addition of silicate minerals increases the expression of the seven target genes

To further elucidate the involvement of the DEGs *lutA_2*, *actP*, *glgC*, *gtaB_3*, *02676*, *levE*, and *glnQ* in the dissolution of biotite or lizardite by C4-10, we measured the relative expression levels of these genes in C4-10 supplemented with biotite or lizardite, comparing them to their expression in the absence of the minerals after 6 and 8 h of incubation (Fig. S6B nad S7B). In the presence of biotite, the relative expression levels of these seven DEGs were significantly upregulated by 0.51- to 40.1-fold after 6 h and 0.58- to 9.4-fold after 8 h of incubation (Fig. S6B). In the presence of lizardite, the relative expression levels of these genes in C4-10 were significantly upregulated by 1.7- to 23.1-fold after 6 h, and 3.3- to 43.8-fold after 8 h of incubation (Fig. S7B). These findings suggest that both biotite and lizardite enhance the expression of these seven DEGs in C4-10, highlighting the critical roles of these genes in the release of Fe from biotite or Mg from lizardite by C4-10.

## DISCUSSION

In this study, we employed a combination of physiological techniques and an integrated multi-omics approach to elucidate the dissolution activity of biotite and lizardite by the mineral-dissolving gram-positive bacterium *P. aryabhattai* C4-10, along with the underlying molecular mechanisms. Our experiments demonstrated that C4-10 enhanced the dissolution of biotite and lizardite through a decrease in pH, the production of mineral dissolution-related metabolites, cell adsorption, and the formation of biofilms on mineral surfaces (Fig. 1 and 2). The genome of C4-10 contains genes associated with organic acid metabolism, cell wall and membrane components related to cell surface adsorption, siderophore and EPS biosynthesis, and biofilm formation, all of which may contribute to mineral dissolution (Fig. S4 and S5). Furthermore, comparative transcriptomic analysis revealed that silicate minerals significantly upregulated the expression of genes related to acid metabolism, biofilm formation, cell wall/membrane metabolism, and transporters in C4-10, which may be implicated in silicate mineral dissolution by this strain.

Organic acids, siderophores, and EPS can enhance dissolution by complexing with ions in solution (8, 12, 24). Moreover, bacterial adhesion to minerals promotes mineral dissolution by elevating proton concentrations, forming mineral surface ion complexes, and catalyzing redox reactions (16, 17). The production of EPS facilitates biofilm formation, thus bringing adsorption sites on the exterior of the cell closer to the mineral surface and generating locally higher concentrations of weathering agents such as siderophores and acids (7, 25). *Bacillus* strains dissolve silicate minerals through the production of organic acids, siderophores, EPS, and their adhesion to minerals (26–29). *P. aryabhattai* SK1-7 was shown to enhance potassium feldspar dissolution via pH reduction and acid production (22). Furthermore, Manimaran et al. (30) demonstrated that *P. aryabhattai* KSBN2K7 produces organic acids that promote silicate mineral dissolution. In this study, we observed a pH drop alongside the production of siderophores and EPS, and cell adsorption and biofilm formation on biotite and lizardite surfaces were observed during the mineral dissolution process of C4-10, indicating that C4-10 significantly enhances the dissolution of both minerals through these physiological activities (Fig. 1 and 2). Notably, the siderophore production by C4-10 in the presence of biotite was detected only in the early stage of the experiment (Fig. 1D), whereas in the presence of lizardite, the siderophore production was detected throughout the whole mineral dissolution process by C4-10 (Fig. 2D). The siderophore production of C4-10 was suppressed in the middle and later stages, correlating with the strain’s increased release of Fe from biotite, as shown by Patel et al. (31), who noted that excess of iron significantly represses siderophore production. These findings suggest that the siderophores produced by C4-10 may play an important role in biotite dissolution during the early stage of the experiment, while those appear to be important for lizardite dissolution throughout the entire experimental duration.

Our understanding of the molecular mechanisms of mineral dissolution is primarily based on studies of gram-negative bacteria (8, 32–34). For instance, Wang et al. (19, 32) reported that *Pseudomonas azotoformans* F77 promotes biotite dissolution through gluconic acid metabolism and pilus-related genes, while Blanco Nouche et al. (33) established that *Caballeronia mineralivorans* PML1(12) utilizes a glucose/methanol/choline oxidoreductase for acidification-based mineral weathering. Moreover, *Pseudomonas* sp. NLX-4 has been observed to promote silicate rock dissolution by upregulating genes associated with siderophore transport, amino acid synthesis, and organic acid metabolism (34). However, there is limited research concerning the molecular mechanisms employed by gram-positive bacteria in dissolving silicate minerals. Liu et al. (23) demonstrated that *B. subtilis* secretes increased amounts of oxidoreductases in the presence of serpentine to facilitate organic acid synthesis and mineral dissolution. Xiao et al. (35) revealed that potassium-bearing rocks induced the production of secreted proteins in *Bacillus mucilaginosus*, speculating that these proteins might expedite potassium mineral dissolution. Furthermore, Yang et al. (22) showed that potassium feldspar induced the upregulation of genes related to pyruvate metabolism, two-component systems, DNA repair, and oxidative stress pathways in the mineral-dissolving strain *P. aryabhattai* SK1-7. In contrast to these studies, our work systematically elucidates a pattern of multi-gene regulation of mineral dissolution in the genus *Priestia* (formerly *Bacillus*). Our study revealed significant upregulation of gene expression and enrichment of metabolic pathways associated with glyoxylate and dicarboxylate metabolism, amino acid biosynthesis, butanoate metabolism, the TCA cycle, and ABC transporters (Fig. 3C), indicating a complex cellular response wherein multiple genes and metabolic pathways contributed to the mineral dissolution process of C4-10. Notably, we identified a conserved set of core genes (*lutA_2*, *actP*, *glgC*, *gtaB_3*, *02676*, *levE*, and *glnQ*) in C4-10, which were significantly upregulated in both mineral systems, and their expression levels positively correlated with the release of Fe or Mg in the presence of biotite or lizardite (Fig. 6 and 7). The upregulated genes are not only involved in organic acid metabolism (e.g., *lutA_2* and *actP*) but also encompass functions related to cell wall synthesis (*gtaB_3*), polysaccharide biosynthesis (*glgC*), and diverse substance transport (*02676*, *levE*, and *glnQ*) (Table 1). Furthermore, the upregulation of *glgC* may be closely linked to the synthesis of biofilm matrix components (36). Biofilm formation enriches cells at the mineral interface and may directly promote mineral dissolution by maintaining a local microenvironment with elevated concentrations of protons and chelators (14, 37). Biofilm formation on mineral surfaces in this study (Fig. 1E and 2E), which was consistent with the upregulated expression of these genes, suggests that C4-10 optimizes its attachment and collective behavior through the modulation of cell wall and storage compound metabolism. Furthermore, the contents of cell-associated Fe and Mg in strain C4-10 increased over time during the mineral dissolution process (Fig. S2), with the genes related to ABC transporters being enriched (Fig. 3C), suggesting that C4-10 may enhance its uptake of nutrients (such as Fe or Mg) released by mineral dissolution, potentially establishing a positive feedback loop. This combined “dissolution-uptake” strategy could confer a competitive advantage for survival in the nutrient-poor mineral environments. While this study provides substantial correlative evidence between gene (*lutA_2*, *actP*, *glgC*, *gtaB_3*, *02676*, *levE*, and *glnQ*) expression and mineral dissolution intensity, the direct functional roles of these genes in mineral dissolution await further validation in the future work. These findings indicate that the mineral dissolution capability of C4-10 arises from a global reprogramming of its metabolic state rather than the activation of a single pathway. Our results expand our understanding of the diverse molecular mechanisms employed by gram-positive mineral-dissolving bacteria. Furthermore, the upregulation of *lutA_2*, *actP*, *gtaB_3*, and *glgC* in C4-10 in the presence of the two minerals may be directly related to mineral dissolution by upregulating organic acid production and cell adsorption, alongside biofilm formation on the mineral surface, whereas the upregulation of *02676*, *levE*, and *glnQ* in C4-10 may relate indirectly to mineral dissolution through enhancing nutrient uptake and bacterial growth.

Ultimately, these findings may have potential applications. Gaining insights into the molecular mechanisms of bacterial silicate dissolution can aid in developing microbe-based technologies for managing agricultural nutrients (3) and strategies for carbon dioxide sequestration (38). The efficient dissolution of two structurally distinct magnesium-bearing silicate minerals by C4-10 indicates its potentially broad-spectrum mineral dissolution capability, presenting a novel candidate strain for leveraging gram-positive bacteria to release nutrients from soil primary minerals or enhance carbon sequestration.

In summary, this study elucidates the physiological and molecular mechanisms by which *P. aryabhattai* C4-10 dissolves biotite and lizardite through the coordinated regulation of multiple genes. These genes (*lutA_2*, *actP*, *glgC*, *gtaB_3*, *02676*, *levE*, and *glnQ*) are involved in various biological processes, including organic acid metabolism, biofilm formation, cell wall synthesis, and substance transport, and their expression was consistently induced in both mineral systems. Correlation analysis reveals the significant role of these genes in the dissolution of biotite or lizardite by C4-10. Notably, C4-10 may dissolve both minerals through similar physiological and molecular mechanisms. Furthermore, *lutA_2*, *actP*, *gtaB_3*, and *glgC* in C4-10 may relate directly to mineral dissolution by regulating organic acid production, cell adsorption, and biofilm formation on the mineral surface, while *02676*, *levE*, and *glnQ* in C4-10 may relate indirectly to mineral dissolution by enhancing Fe and Mg uptake and bacterial growth. This suggests that C4-10 employs a common molecular strategy to interact with distinct silicate minerals, and the mineral dissolution capability of C4-10 arises from a global reprogramming of its metabolic state. Our findings not only deepen the understanding of mineral dissolution mechanisms in gram-positive endospore-containing bacteria but may also provide a theoretical foundation for enhancing the environmental application potential of such strains.

## MATERIALS AND METHODS

### Isolation of thermotolerant mineral-dissolving bacteria

Rhizosphere soil from *C. camphora* collected from Zijin Mountain in Nanjing, China, was utilized for bacterial isolation. Bacterial isolation and mineral dissolution experiments were conducted using BHm medium supplemented with biotite, following the methodology outlined by Huang et al. (10), with some modifications. Prior to isolation, the soil suspension underwent a 30-min treatment in a water bath at 80°C to eliminate non-thermotolerant bacteria. Soil samples (1.0 g, wet weight) were introduced into Erlenmeyer flasks containing 100 mL of sterile 0.85% NaCl solution and agitated at 180 rpm for 30 min to facilitate the detachment of bacteria from soil particles. Serial 10-fold dilutions of the sample suspensions were plated onto 1/5-strength LB medium (per liter of distilled water: 5 g yeast extract, 10 g tryptone, and 10 g NaCl, adjusted to a pH of 7.0) agar plates, with 0.1 mL of bacterial suspension spread onto each plate. The plates were incubated at 37°C for 3 days. All bacterial colonies were subsequently picked from the plates for further study of biotite dissolution. Based on the effectiveness of Fe and Al release from biotite, strain C4-10 was selected for its superior capacity to release significant levels of Fe and Al and for its associated mechanisms of mineral dissolution. Strain C4-10 was identified through 16S rRNA gene sequencing and whole-genome sequencing analyses (10).

### Silicate minerals, media, and culture conditions

Biotite and lizardite, both prevalent silicate minerals, were utilized in this study. The elemental compositions of these minerals were as follows: biotite contained 39.90% SiO_2,_ 18.98% Al_2_O_3_, 14.75% Fe_2_O_3_, 13.69% MgO, 9.12% K_2_O, 0.28% Na_2_O, and 0.07% CaO, while lizardite comprised 44.35% SiO_2_, 42.85% MgO, 3.93% Fe_2_O_3_, 1.84% Al_2_O_3_, 1.52% CaO, 0.09% Na_2_O, 0.02% K_2_O, and 0.02% TiO_2_. In biotite and lizardite, Fe/Si and Mg/Si serve as principal structural elements, respectively. The concentrations of dissolved Fe/Si and Mg/Si in the medium served as indicators of the dissolution of biotite or lizardite. The minerals, with particle sizes ranging from 75 to 150 μm, were cleaned according to the method described by Sheng et al. (39). The BHm solution utilized for the mineral dissolution experiments consisted of 0.02 g L^−1^ KCl, 0.15 g L^−1^ MgSO_4_·7H_2_O, 0.08 g L^−1^ NaH_2_PO_4_·2H_2_O, 0.09 g L^−1^ Na_2_HPO_4_·2H_2_O, 0.065 g L^−1^ (NH_4_)_2_SO_4_, 0.1 g L^−1^ KNO_3_, 0.02 g L^−1^ CaCl_2_, and 2 g L^−1^ glucose and was maintained at a pH of 7.0 (40). Strain C4-10 was cultivated in LB medium (pH 7.0) and incubated for 16 h at 37°C on a rotary shaker operating at 180 rpm.

### Biotite dissolution capacity and mineral dissolution-related physiological activity of C4-10

The biotite dissolution experiment involving C4-10 was conducted in accordance with the protocols established by Wang et al. (32) and Dong et al. (41) with certain modifications. C4-10 was cultured in liquid LB medium, harvested via centrifugation, washed, and subsequently resuspended in sterile distilled water to achieve a final concentration of 10^8^ cells mL^−1^. Triplicate flasks, each containing 60 mL of sterilized BHm and 0.6 g of biotite, were inoculated with 1.5 mL of the bacterial suspension. The flasks were incubated at 37°C on a rotary shaker at 180 rpm for varying time intervals of 4, 8, 12, 16, 20, 24, 36, and 48 h. At these intervals, measurements were taken for the concentrations of dissolved Fe and Si, pH, cell counts, siderophore production, as well as organic acid and EPS concentrations. Fe and Si concentrations were analyzed using an inductively coupled plasma optical emission spectrometer (ICP-OES) (Optimal 2100 DV, PerkinElmer). Cell counts in the medium were determined using a 10-fold dilution method (32). Cell counts on the mineral surfaces were evaluated using ninhydrin colorimetry (42). Cell-associated Fe contents were quantified after 12 and 24 h of incubation. pH was measured with a pH meter (PHS-3CT). Organic acid and EPS concentrations in the medium were assessed by high-performance liquid chromatography and the phenol-sulfuric acid method, respectively, as outlined by Chen et al. (42). A detailed analysis of cell counts on the mineral surface, production of siderophores in the medium, and cell-associated Fe contents is provided in the supplemental materials. Biofilm formation by C4-10 on the biotite surface was visualized using fluorescence microscopy after 24 h of incubation, as described by Sheng et al. (43). Mineral particles were rinsed three times with a 0.85% NaCl solution and gently removed from glass slides. Cells attached to biotite were stained with the nucleic acid stain SYTO9 (Thermo Fisher Scientific, USA) and subsequently washed with a 0.85% NaCl solution. After air-drying at room temperature, the samples were examined using a fluorescence microscope (Axiovert-100 MBP Microscope, Zeiss) equipped with AxioVision 4.2 software (Zeiss).

### Complete genome sequencing of strain C4-10

The genome DNA of C4-10 was prepared as outlined by Wilson (44). Subsequent sequencing was performed using Illumina HiSeq technology by Shanghai BIOZERON Biotechnology Co., Ltd. (Shanghai, China). During preprocessing, reads containing poly-N sequences, adapters, errors, low-quality reads, and small fragments (length <50 bp) were eliminated from the raw data set. High-quality reads were assembled using SOAPdenovo (version 2.04). Gaps were filled and bases corrected using GapCloser (version 1.12) software (19). Genome annotation was carried out using the NCBI Prokaryotic Genome Annotation Pipeline (45). Functional classification involved aligning predicted proteins to the Clusters of Orthologous Groups database (46).

### Sample collection, RNA extraction, and transcriptome sequencing

For transcriptome analysis, two experimental groups (C4-10 with or without biotite) were created. The BHm medium, with or without biotite, was employed for culturing C4-10 to investigate differential gene expression. C4-10 was cultivated in liquid LB medium, followed by centrifugation, washing, and resuspension in sterile distilled water to achieve a final concentration of 10^8^ cells mL^−1^. Triplicate flasks, each containing 60 mL of sterilized BHm (with or without 0.6 g biotite), were inoculated with 0.5 mL of the bacterial suspension. The flasks were incubated at 37°C on a rotary shaker at 180 rpm. After 8 h of incubation, during which the Fe concentration in the medium significantly increased in the presence of C4-10, total RNA was extracted from the samples using a Bacterial RNA Kit (Omega Bio-tek, USA) according to the manufacturer’s instructions. The quality and concentration of RNA were assessed with a 2100 Bioanalyzer (Agilent Technologies, USA) and a NanoDrop ND-2000 (Thermo Fisher Scientific). Following RNA extraction, mRNA was enriched using a Ribo-Zero Magnetic Kit (Epicenter, USA), fragmented into short segments, and reverse-transcribed into cDNA using random primers. Second-strand cDNA was synthesized using DNA polymerase I. The cDNA fragments were purified with a QiaQuick PCR extraction kit, end-repaired, poly(A)-added, and ligated to Illumina sequencing adapters. The ligation products were size-selected via agarose gel electrophoresis, PCR amplified, and sequenced using Illumina HiSeq2500 by Gene Denovo Biotechnology Co. (Guangzhou, China).

### Differentially expressed genes and functional enrichment

The raw paired-end reads obtained from high-throughput sequencing underwent trimming and quality control using SeqPrep (https://github.com/jstjohn/SeqPrep) and Sickle (https://github.com/najoshi/sickle) with default parameters. Clean reads were generated by removing adaptor sequences and eliminating reads containing more than 10% of unknown nucleotides and more than 50% of low-quality (*Q* value ≤20) bases from the raw data. The Bowtie2 short-read alignment tool Bowtie2 (47) was employed to map reads to the ribosomal RNA database, and rRNA-mapped reads were subsequently excluded from further analysis. Remaining reads were then employed in the assembly and analysis of the transcriptome. rRNA-removed reads of each sample were mapped to the C4-10 reference genome using TopHat2 (version 2.0.3.12) (48). DEGs between the two groups were identified by calculating the expression level of each transcript based on the fragments per kilobase of exon per million mapped reads using the edgeR package (http://www.r-project.org/). Genes exhibiting a fold change of >1.2 and a *P* value of <0.05 were categorized as significant DEGs. The DEGs were subjected to GO functional analysis and KEGG pathway analysis to elucidate differences in gene functions and metabolic pathways among the samples. To assess the enrichment of DEGs compared with all identified genes within each GO and KEGG category, a two-tailed Fisher’s exact test with standard false discovery rate control methods was employed for correction, considering a corrected *P* value of <0.05 statistically significant for both GO categories and KEGG pathways.

### Short-term biotite dissolution activity and expression of target genes

To evaluate the correlations between Fe concentrations and expression levels of seven biotite-induced significantly upregulated genes (*lutA_2* and *actP* associated with acid metabolism, *glgC* associated with biofilm formation, *gtaB_3* involved in cell wall component, and *02676*, *levE*, and *glnQ* involved in transporters), a short-term biotite dissolution experiment was conducted, adhering to the detailed protocols described earlier. The flasks were incubated at 37°C on a rotary shaker at 180 rpm at 5, 6, 7, 8, and 9 h. At each incubation time, the dissolved Fe concentrations were analyzed with ICP-OES, and gene expression levels were quantified using RT-qPCR.

Bacterial cells were collected by centrifugation at 9,200 × *g* for 5 min at 4°C. Total RNA of C4-10 was extracted using AG RNAex Pro Reagent (AG21101; Accurate Biotechnology Co., Ltd, Hunan, China), according to the manufacturer’s instructions. Quantitative reverse transcription was then performed on 1 μg of total RNA using the Evo M-MLV RT Mix Kit with gDNA Clean for qPCR based on the manufacturer’s protocols. RT-qPCR was performed on an Applied Biosystems 7500 real-time PCR system using SYBR Premix Ex Taq (TaKaRa, Japan). Gene-specific primers were designed using Primer Premier 5.0. The primers used for RT–qPCR are listed in Table 2, and the DNA sequences of the target genes are presented in Fig. S8 to S15. The relative expression of the seven target genes (*lutA_2*, *actP*, *glgC*, *gtaB_3*, *levE*, *02676*, and *glnQ*) was normalized using *recA* (encoding recombinase) as an internal reference gene. Reactions were performed in triplicate, with amplification procedures including 40 cycles of 95°C for 5 s, followed by 55°C for 15 s, and 72°C for 30 s. The 2^−ΔΔCT^ method was used to calculate the relative expression levels (19).

**TABLE 2:** Primers used for RT-qPCR analysis in this study

Primers	Sequence (5′−3′)	Primers	Sequence (5′−3′)
recAF	GAAGCGGCGCAGTAGATATT	recAR	CACGTGAGAGTCTCCCATTTC
glnQF	CCTACCTCTGCTCTTGAT	glnQR	TACCATCGTCATTCCTTCT
gtaB_3F	ACCGATTATACGAAGTGAATG	gtaB_3R	CTGAATAGCATCCGTTAGC
glgCF	GTAGAGCAGTCCGTGTTA	glgCR	CGTCTGGAACTTCAATGTC
02676F	TTGTCAATGGAGCCGTTA	02676R	CCGTTATGAGGAAGCGTAT
actPF	TGCTGGTTGGACTATTCA	actPR	GCCTAGAGGAATGGAGATAA
lutA_2F	GCTGTTAGTGGAATACGA	lutA_2R	AATGACACGAATCTTGATAC
levEF	TGCCTATCGAGACAGTAA	levER	CGGTAACGCTTACAGATT

### Impact of biotite on the expression of target genes

To evaluate the expression levels of the seven target genes (*lutA_2*, *actP*, *glgC*, *gtaB_3*, *02676*, *levE*, and *glnQ*) in the inoculation of C4-10 with or without biotite, triplicate flasks containing 60 mL of sterilized BHm, with or without 0.6 g biotite, were inoculated with 1.5 mL of a bacterial suspension as previously described. The flasks were cultured at 37°C for 6 and 8 h to assess the relative expression levels of these target genes in the presence of biotite compared to their levels in the absence of the mineral. RT-qPCR analysis was performed in triplicate to evaluate the impact of biotite on these genes in C4-10 as described earlier.

### Lizardite dissolution capacity, mineral dissolution-related physiological activity, and expression of target genes of C4-10

To better understand the effect of C4-10 on silicate mineral dissolution and the underlying mechanisms, the lizardite dissolution capacity, mineral dissolution-related physiological activity (including pH, siderophore and EPS production in the medium, cell adsorption, and biofilm formation on the mineral surface), cell-associated Mg contents, and expression of the seven target genes (*lutA_2*, *actP*, *glgC*, *gtaB_3*, *02676*, *levE*, and *glnQ*) of C4-10 were determined as described above with some modifications. Strain C4-10 was cultivated in a modified BHm medium devoid of MgSO_4_·7H_2_O. The dissolved Mg concentrations, pH, siderophore production, cell counts in the medium and on the lizardite surface, EPS concentration, cell-associated Mg contents, and the formation of biofilm on the lizardite surface were evaluated at various incubation times. To ascertain the correlations between Mg concentrations and expression levels of the seven target genes in the process of lizardite dissolution by C4-10, short-term lizardite dissolution experiment of C4-10 was performed, following the detailed protocols as described above. The flasks were incubated at 37°C on a rotary shaker at 180 rpm for 4, 5, 6, 7, and 8 h. The dissolved Mg concentrations were analyzed using ICP-OES at different incubation times. The expression levels of the target genes in C4-10 with or without the inoculation of lizardite at various times were determined in triplicate through RT-qPCR analysis.

### Statistical analysis

Two-tailed *P* values from unpaired *t*-tests were employed to compare the concentrations of Fe or Mg and Si released from the minerals by C4-10 to those in the uninoculated controls. Additionally, cell counts, pH values, and the concentrations of organic acid, siderophore, and EPS, and relative expression levels of genes in the presence of C4-10 were assessed. Pearson correlation coefficient analysis was conducted to examine the relationships between gene expression and Fe/Mg concentrations. All statistical analyses were carried out using GraphPad Prism 9.3.

## Data Availability

Raw sequences have been deposited in the GenBank database under accession number GCA_039631325.1 for *Priestia aryabhattai* C4-10, ABDD91_RS20515 for gene recA (recombinase RecA), ABDD91_RS01610 for gene glnQ (amino acid ABC transporter ATP-binding protein), ABDD91_RS07970 for gene levE (PTS system mannose/fructose/N-acetylgalactosamine-transporter subunit IIB), ABDD91_RS07585 for gene gtaB_3 (UTP-glucose-1-phosphate uridylyltransferase GalU), ABDD91_RS24435 for gene glgC (glucose-1-phosphate adenylyltransferase), ABDD91_RS13425 for gene 02676 (GntP family permease), ABDD91_RS04670 for gene actP (cation acetate symporter), and ABDD91_RS13400 for gene lutA_2 (glycolate dehydrogenase iron-sulfur subunit). The raw transcriptome sequencing data generated in this study have been submitted to the NCBI SRA (https://submit.ncbi.nlm.nih.gov/subs/sra/) and are accessible under the accession number PRJNA1312293.

## SUPPLEMENTAL MATERIAL

Supplemental material aem.02554-25-s0001.pdf Supplemental methods and Fig. S1 to S15.

ASM does not own the copyrights to Supplemental Material that may be linked to, or accessed through, an article. The authors have granted ASM a non-exclusive, world-wide license to publish the Supplemental Material files. Please contact the corresponding author directly for reuse.
