# Supplementary material for: Silicate minerals enhance the expression of genes related to mineral dissolution by Priestia aryabhattai strain C4-10
Source: Appl Environ Microbiol. 2026 Jan 26;92(2):e02554-25. doi: 10.1128/aem.02554-25 (PMC12915317; doi:10.1128/aem.02554-25)
Supplement: Supplemental material — Supplemental methods and Fig. S1 to S15. [file aem.02554-25-s0001.pdf]

Silicate Minerals Enhance the Expression of Genes Related to Mineral Dissolution by *Priestia aryabhattai* Strain C4-10

Qi Sheng, Xin-Yi Zheng, Si-Han Yang, Wen Dong*, Lin-Yan He, Xia-Fang Sheng*

College of Life Sciences, Nanjing Agricultural University, Nanjing 210095, China

Supporting Materials and Methods

Determination of cell counts on the mineral surfaces. Cell counts on the biotite surfaces were determined using ninhydrin colorimetry as described by Chen et al. (2016). Briefly, all of the mineral particles with adhered bacteria were added to tubes and washed with sulfuric acid solution (pH 2) to remove loosely held cells to estimate the total number of cells on the mineral surface. The cells from the mineral particles that contained adhered bacteria were then digested in 5 mL of 0.5 M NaOH in a boiling water bath for 25 min to digest the bacterial cells. After cooling, the pH of the digest was adjusted to pH 7.0 with 0.5 M HCl, and its volume was adjusted to 5.0 ml with sterile distilled water. Then, 1 mL of digest from each sample was transferred to a clean tube, and 0.5 mL ninhydrin reagent was added. The tubes were put in boiling water for 20 min. After cooling, 2.5 mL of 50% (vol/vol) ethanol was added to each tube and thoroughly mixed by vortexing. The absorbances of the solutions in the tubes were then measured at 570 nm within 1 h. To correlate the cell concentrations of the strains with measured absorbances, calibration curves were prepared. Standard curves showing plots of the optical density from the ninhydrin reaction of each of the cell digests against the corresponding cell concentration for the strains were made.

In order to correlate the cell concentrations of strain C4-10 with measured absorbances, one calibration curve was prepared. For this purpose, 1 ml of strain C4-10 was diluted with sterile distilled water (the cells could be viable in the distilled water), and 0.1 ml of cell suspension was plated onto LB agar plate to determine the total bacterial number. The plates were incubated at 30°C for 7 days. The number of colonies on each plate was then counted, and each of the counts was converted to the number of CFU per milliliter in the original culture.

Standard curve showing plots of the optical density (OD) from the ninhydrin reaction of the cell digests against the corresponding cell concentration for strain C4-10 was made. The ninhydrin reaction was run on 0.05, 0.1, 0.2, 0.3, 0.5, 0.8, and 1 ml of culture suspension. The suspension was centrifuged at 4,000 rpm for 5 min to separate the cells from the medium. The cells were then digested, and the ninhydrin reaction was run on the digest to determine the corresponding peptide concentration (Allen, 1981). Each sample was analyzed in triplicate, and relative standard deviation was determined. The cell concentrations of the suspension were determined by plating.

Determination of the production of siderophores in the medium. The production of siderophores in the medium was determined in terms of siderophore units (%) using the chromeazurol S (CAS)-liquid assay (Machuca and Milagres 2003; Sheng et al., 2024). The sample (s) and reference (r) absorbances at 630 nm were measured after different incubation times at room temperature. The percentage of iron-binding of siderophores was calculated by subtracting the sample absorbance values from the reference value. Siderophore units are defined as [(Ar–As/Ar)] ×100 = % siderophore units (Machuca and Milagres 2003). Percentages of siderophore units less than 10 were considered negative, and in this case, no change in the blue color of the CAS solution was observed. Ar was determined using the CAS assay solution mixed with Bushnell Haas medium.

Determination of cell-associated Fe or Mg contents of the growing cells. Cell-associated Fe or Mg contents of the growing cells in the presence of biotite or lizardite were determined after 12 h and 24 h of incubation. The harvested culture medium containing mineral particles and cells was centrifuged at 1,000 rpm for 1 min to remove mineral particles, then the remaining culture medium containing cells was further centrifuged at 8,000 rpm for 5 min to remove the supernatant. The cell pellets were washed with sterilized physiological saline (0.85% NaCl) for five times and centrifuged at 1,000 rpm for 1 min after each wash until no mineral particles are visible. The samples were centrifuged at 8,000 rpm for 5 min to remove the supernatant. The cell pellets were freeze-drying and digested in 1 mL of H_2O_2 in a boiling water bath for 2 h to digest the bacterial cells. After cooling, the digest solution is filtered through a 0.45 μm filter and add an equal volume of 10% HNO_3. The cell-associated Fe or Mg contents in the samples were determined with ICP-OES. All the experiments were carried out in triplicate.

References:

Allen G. 1981. Methods for the detection of peptides, p 139–141. In Work TS, Burdon RH (ed), Laboratory techniques in biochemistry and molecular biology. Elsevier North Holland Biomedical Press, Netherlands.

Chen W, Luo L, He LY, Wang Q, Sheng XF. 2016. Distinct mineral weathering behaviors of the novel mineral-weathering strains *Rhizobium yantingense* H66 and *Rhizobium etli* CFN42. Applied and Environmental Microbiology, 82, 4090–4099.

Han H, Wang Q, He LY, Sheng XF. 2018. Increased biomass and reduced rapeseed Cd accumulation of oilseed rape in the presence of Cd-immobilizing and polyamine-producing bacteria. J Hazard Mater, 353, 280–289.

Machuca A, Milagres AMF. 2003. Use of CAS-agar plate modified to study the effect of different variables on the siderophore production by *Aspergillus*. Lett Appl Microbiol 36, 177–181.

Sheng Q, Li L, Dong W, He LY, Sheng XF. 2024. Impact of the iron-response regulator genes on the release of iron and aluminum from biotite by *Rhizobium pusense* S41. Geomicrobiol J 41, 298–307.

Supporting Figures

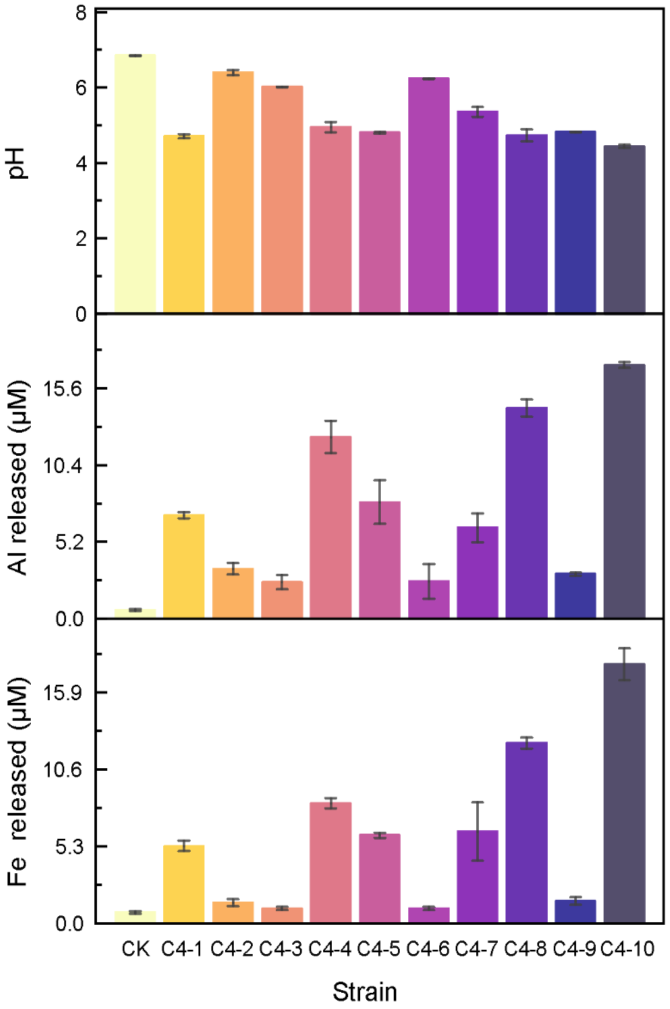

Fig. S1. Fe and Al concentrations and pH in the medium supplemented with biotite (0.3 g) in the presence of the thermotolerant bacterial strains (C4-1 to C4-10) cultured on a rotary shaker at 160 rpm for 24 h at 37°C. Error bars represent ± one standard deviation (*n*=3).

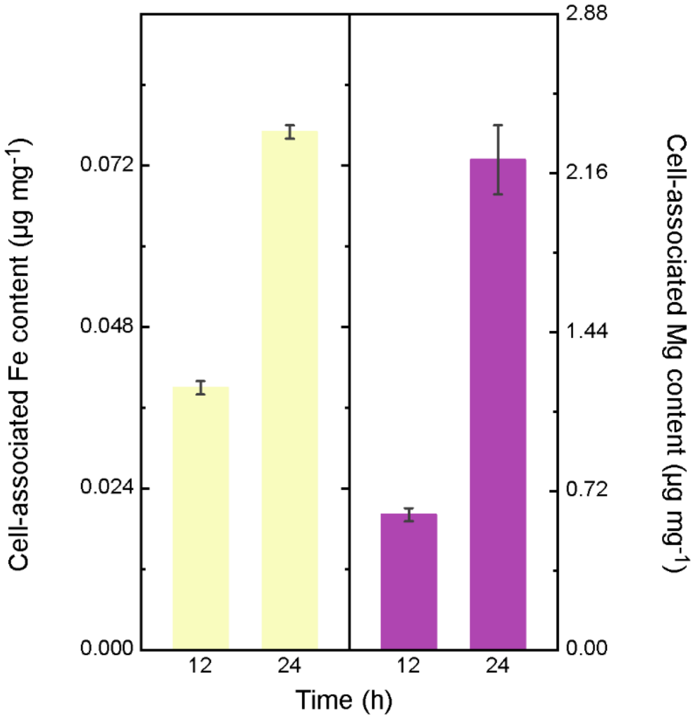

Fig. S2. Cell-associated Fe or Mg contents of C4-10 cultured in the presence of biotite or lizardite after 12 h and 24 h of incubation. Error bars represent ± one standard deviation (*n*=3).

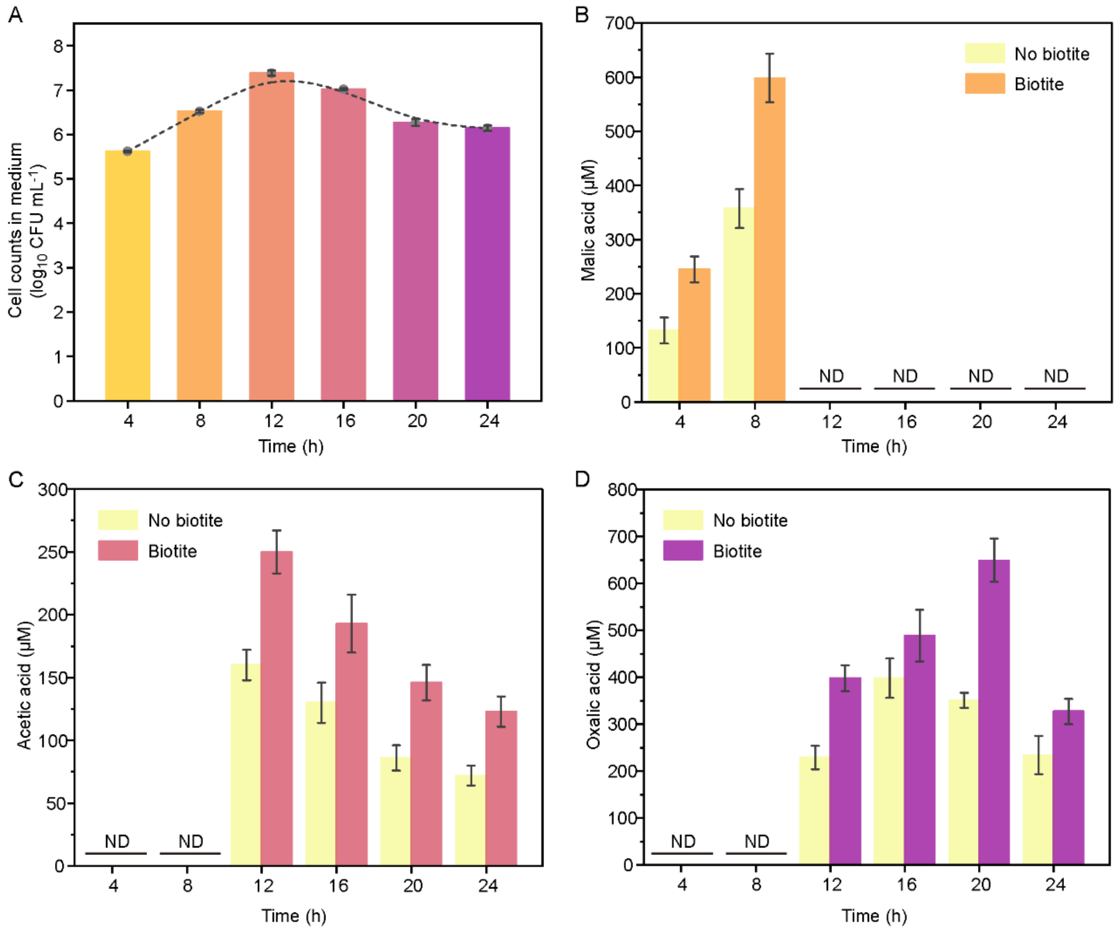

Fig. S3. Cell counts in the absence of biotite (A) and concentrations of malic acid (B), acetic acid (C), and oxalic acid (D) in the C4-10-inoculated medium in the presence and absence of biotite. Error bars represent ± one standard deviation (*n*=3).

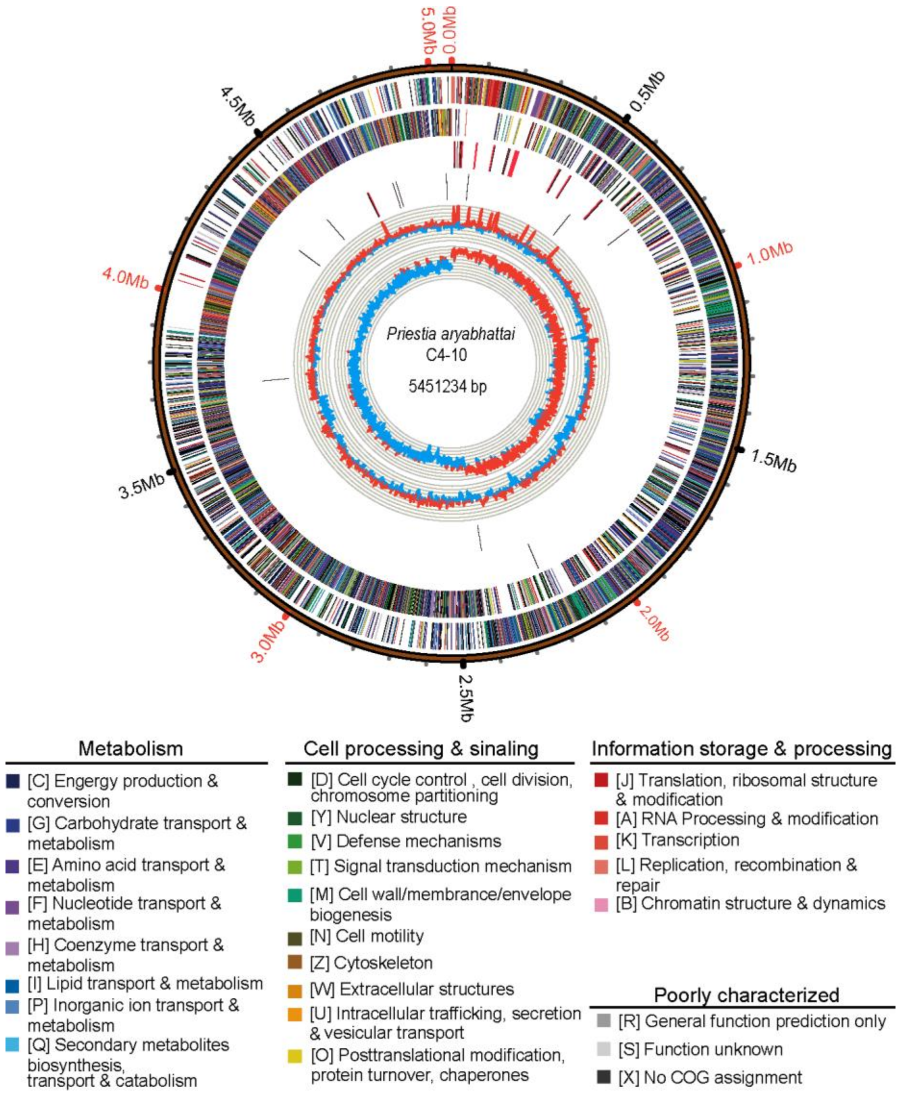

Fig. S4. Genetic map of C4-10

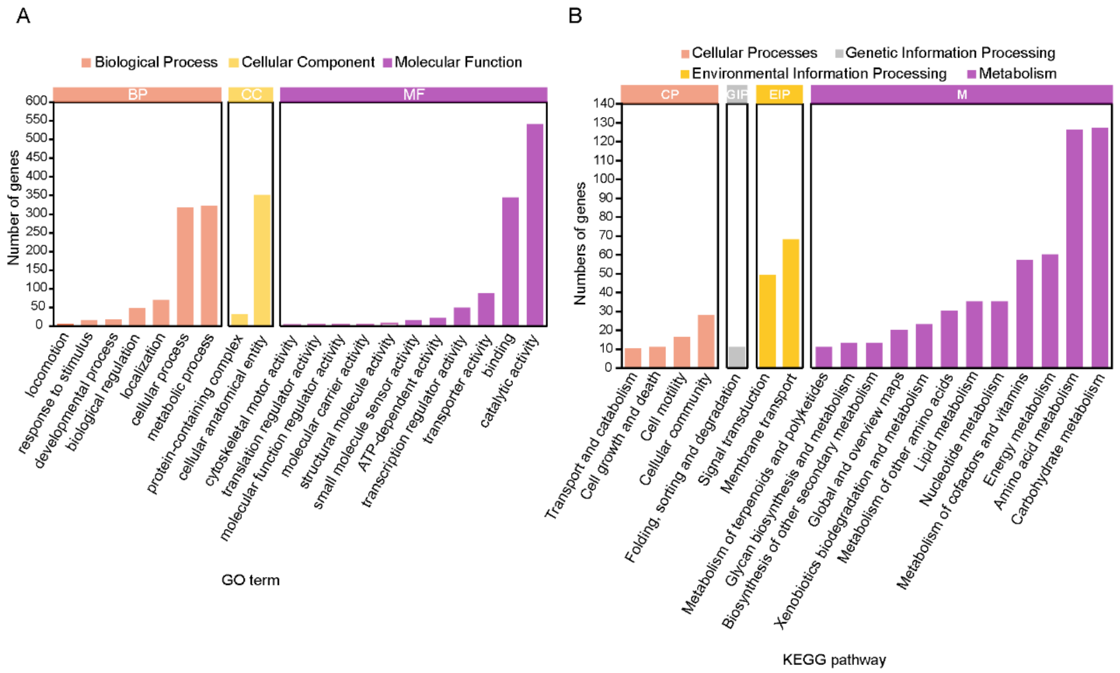

Fig. S5. Transcriptomic analysis of C4-10 cultivated in the BHm in the presence of biotite compared with that in the absence of the mineral. Go annotation (A) and KEGG pathway annotation (B) analyses of upregulated DEGs.

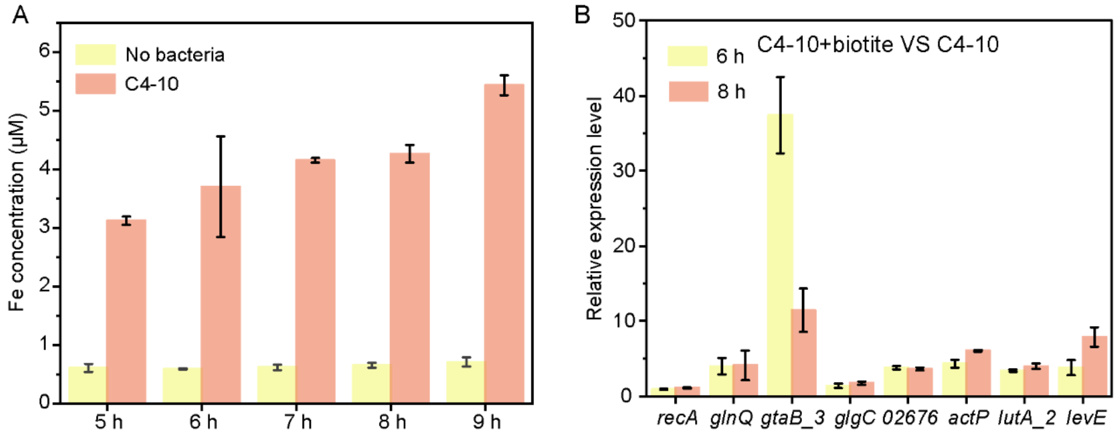

Fig. S6. A. Fe concentrations in the C4-10-inoculated medium in the presence of biotite. B. Relative expression levels of mineral dissolution-related genes (*glnQ*, *gtaB_3*, *glgC*, *02676*, *actP*, *lut_2*, and *levE*) in the presence of biotite compared to their levels in the absence of the mineral, analyzed by RT-qPCR at 6 h and 8 h of incubation. Error bars represent ± one standard deviation (*n*=3).

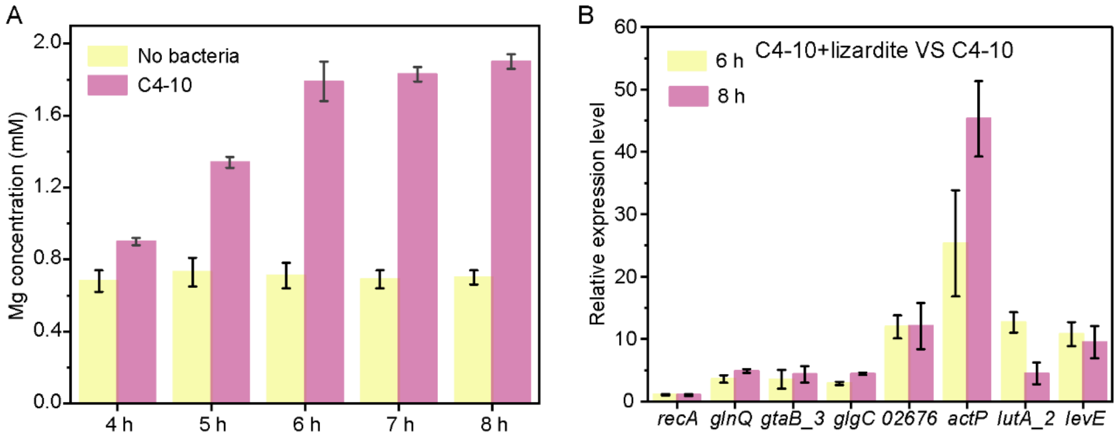

Fig. S7. A. Mg concentrations in the C4-10-inoculated medium in the presence of lizardite. B. Relative expression levels of mineral dissolution-related genes (*glnQ*, *gtaB_3*, *glgC*, *02676*, *actP*, *lut_2*, and *levE*) in the presence of lizardite compared to their levels in the absence of the mineral, analyzed by RT-qPCR at 6 h and 8 h of incubation. Error bars represent ± one standard deviation (n=3).

> ABDD91_RS20515 *recA*

GTGAACGATCGTCAAGCAGCCCTTGATATGGCTTTAAAGCAAATTGAAAAGCAATTTGGTAAAGGTT

CAATTATGAAATTAGGTGAACAAACGGAAAAAAGAATTTCTACAATTCCAAGTGGTTCATTAGCATT

AGATATAGCTTTAGGCGTAGGTGGATATCCACGTGGACGTGTGGTGGAAGTATATGGCCCAGAAAGC

TCAGGTAAAACAACAGTTGCTCTTCACGCGATTGCAGAAGTTCAACAGCAGGGCGGACAGGCTGCAT

TTATCGATGCGGAGCACGCGTTAGATCCTGTATATGCTCAAAAATTAGGTGTTAATATTGATGAGCTG

TTATTATCTCAGCCTGATACAGGAGAACAAGCTTTAGAAATCGCTGAAGCTTTAGTTCGAAGCGGCG

CAGTAGATATTATTGTTGTTGACTCAGTAGCAGCGTTAGTGCCAAAAGCGGAAATTGAAGGAGAAAT

GGGAGACTCTCACGTGGGTCTACAAGCTCGTTTAATGTCTCAAGCATTGCGTAAACTATCTGGAGCT

ATTAACAAGTCTAAAACAATCGCTATCTTTATTAACCAAATTCGTGAAAAAGTCGGCGTAATGTTTG

GTAACCCTGAAACAACTCCTGGTGGACGTGCGCTTAAATTCTACTCTTCAGTGCGTCTAGAAGTGCGT

CGTGCAGAGCAGTTAAAGCAAGGAAATGATATCGTAGGTAACAAAACAAGAATTAAAGTTGTGAAA

AATAAAGTAGCTCCGCCTTTCCGTGCTGCCGAAGTAGATATTATGTACGGAGAAGGTATTTCAAAAG

AGGGTGAAATTTTGGATATCGCTTCTGAACTAGATATTGTCCAAAAAAGTGGATCTTGGTATTCATAT

AATGACGAGCGTCTAGGTCAAGGCCGTGAAAATGCAAAGCAATTCTTAAAAGAAAATACTGATATTC

GTCAGGAAATTGCGGGACAAGTGCGTGAACATCATGGTTTAGACCAAGATGGAGAGCCAGCTCCTG

AGGATGACGATCAAGGCGATTTAAATATTTAA

Fig. S8. DNA sequence of *recA*

> ABDD91_RS01610 *glnQ*

ATGATTGTTTTTGACGGAGTGAACAAATATTACGGTGATTTTCACGTATTAAAAGATATCAATTTAAC

GATTAAAAAAGGTGAAGTTGTCGTCGTAATTGGACCTTCGGGTTCGGGTAAAAGCACGATGCTGCGA

TGCATAAATTATTTAGAAACAATATCTAACGGACAATTGACGGTAAATAATATATTAGTTGCAGATA

AAAAAACAAATATTAACAAACTGCGCCGCAATATAGGTATGGTGTTTCAGCATTTTTATTTATATCCG

CACAAAACGGTTTTAGAAAATATCACCTTAGCGCCTATGAAAGTACTGGGACAGTCGTCAGAAGAAG

CTAAGAAAACAGCTCTATACTATTTGGATAAAGTAGGAATCGGTGAAAAAGCCAACGCCTACCCATC

CCAGCTTTCAGGCGGCCAGCAGCAGCGTGTAGCCATTGCGAGGGGACTTGCGATGAAACCAGAGATT

ATGCTTTTTGATGAGCCTACCTCTGCTCTTGATCCAGAAATGATTGGAGAAGTGCTGGATGTCATGAA

AACATTGGCTAAAGAAGGAATGACGATGGTAGTTGTAACTCATGAAATGGGGTTTGCTAAAGAAGT

AGCAGACCGCATTGTATTTATGGATCAAGGAAAAATCTTAGAAGAGGCAACTCCGGCTGAGTTTTAT

GAAAACCCGCAAGAAGAAAGGGCACGTTTATTTCTTAGCCGTATTTTAAATCATTAA

Fig. S9. DNA sequence of *glnQ*

> ABDD91_RS07585 *gtaB_3*

ATGCAGACTATAAAAAAAGCAGTTATACCAGCAGCAGGGTTAGGTACTCGTTTTTACCCGTTACAA

AATCTATCCCAAAAGAAATGCTTCCAATTGTAAATAAGCCCGTCATTCAATTTATTGTGGAAGAAGC

CTTAAAATCAGGCATCGAAGATATTTTAATTGTTACAGGAAACGGAAAACAAGCGATTGAAAATCAT

TTTGACCATAATATTCAATTAGAACACTTGCTTCATCAAAAAGGAAAGACCGAACTATTAGAGGAAA

TGGAGCATATCTCTGAACTAGCGAATATTCATTACGTTCGTCAAAAAGAAATGAAGGGCCTTGGCCA

TGCGATTGGCTGTGCTCGACAATTTATTGGAAACGAACCGTTTGCAGTTTTACTTGGAGATGACTTAA

CAGACCCAGATCAGCCTTGTTTAAAACAGCTGATTGACCAGTATACCCAAACGGGATCGTCGGTTAT

CGGCGTGCAGCGCGTAGAGGAAGAAGCCGTTCATCGTTACGGGATTATAGATCCAAAAGTAAATAA

AAACCGATTATACGAAGTGAATGGTTTTGTAGAAAAACCATCAGTCGAAGAAGCTCCGTCTAATTTA

GGAATTATTGGACGCTATGTATTTACACCTGACATTTTTGATTATCTTGAAACGCAGGAAGCTGGAAA

AGGTGGGGAGATTCAGCTAACGGATGCTATTCAGTGTATGAATATAGACCGTTCTATTTATGCTTACG

AATTCGAAGGCGAACGTTATGATGCAGGGGAAAAATTAGATTTTATTTTTACGACCCTTGCTTTTGCG

TTAAAAGATGAAGAATTAAAAGCACCTCTTTTAACTAAATTCAAAGAGCTGATCAACAAGGAAGAA

CAAAAAGAAAAAATTGCTGTACAAATTGGAAAATAA

Fig. S10. DNA sequence of *gtaB_3*

> ABDD91_RS24435 *glgC*

ATGTTAAAAAAGAAATGCGTAGCGATGTTATTGGCAGGAGGAAAAGGTAGTCGGCTAAGTTCGCTTA

CAAAAAATCTAGCAAAGCCAGCTGTACCATTTGGTGGTAAGTACCGAATTATTGATTTTGCTTTAAGT

AATTGTACAAATTCAGGTATTGAAACAGTAGGTGTATTAACACAATATCAACCGCTAGTTCTCAATTC

ATACATTGGAATTGGAAGCGCTTGGGATTTGGACCGACGGAATGGTGGCGTCACAGTATTGCCTCCA

TACGCGGAGTCAGATGGAGTAAAGTGGTATAAAGGCACGGCTAGCGCTATTTATGAAAATTTAAACT

ATTTGACACAATATGATCCTGAGTACGTACTGATTCTATCAGGTGATCACATTTATAAAATGAACTAT

GAAAATATGCTTGATTATCATATAAACAAAGAAGCAGATGTAACGATTTCTGTTATTGAAGTACCTT

GGGAAGAAGCGAGCCGTTTTGGTATTTTAAATACAAATAGTGACCTAGATGTAATGGAGTTCGATGA

AAAGCCACAGCGTCCTAAAAATAATCTAGCTTCTATGGGGATTTATATTTTTAAATGGAGTATTTTAA

AAGAATATTTAGAAATGGATGCACGCAATCCATACTCAAGCCATGATTTTGGTAAAGATGTTATTCC

GCTTTTACTAGATGAAAAGAAAAAGCTAATTGCATATCCATTCCAAGGTTATTGGAAAGATGTAGGA

ACTGTAAAAAGCTTATGGGAAGCGAATATGGATTTGTTATGTGATAAAGACGAGCTTAATTTATTTG

ACTCTTCATGGAAAGTATACTCTGTTAACCCTAATCAGCCTCCTCAATATATTGCGCCAAACGCCTGC

GTGGTGGAGTCATTAGTAAATGAAGGGTGCGTTGTCGAAGGAAACGTAGAGCAGTCCGTGTTATTTC

CAGGTGTACAAATCGGTTCAGGTTCTGAAGTTAAGAAAACGGTTGTCATGCCTACTGCAAAGATAGG

CTCTAACGTATATATTGAAAACGCAATTGTTCCATCGGACATTGAAGTTCCAGACGGTACTATCATTC

GACCGACCAAAGGAAGCGAAGAAGTAATTCTTGTTACTCAAGAATTGATTGAATCAGTTGCTAAATG

CATTTAA

Fig. S11. DNA sequence of *glgC*

> ABDD91_RS13425 *02676*

ATGGATTTGCTGATTATTTTGCTGTCTCTGGGGCTTTTGATGCTGGTTGCTTACCGCGGATTTTCAGTT

ATTTTGTTTGCTCCACTTTGTGCGCTGCTAGCCGTTATTTTAACGGAACCAAGCTATGCACTCCCATTT

TTCTCAAACGTTTTTATGGAGAAAATGGTTGGATTTATTAAAAACTACTTCCCTGTCTTTCTATTAGG

AGCAGTCTTCGGGAAGATGGTTGAAATGTCTGGAGTCGCTGAATCGATTGCGAAAACAATTGTTAAA

GTCGTGGGGGCTAAACGTGCGATCTTGGCGATTGTTTTAATGGGAGCAATCTTAACCTACAGCGGCG

TAAGTTTATTCGTTGTTGCGTTTGCAGTTTATCCGTTTGCGGCTAACTTATTCCGCGAAGCGAATATTC

CAAAACGATTAGTACCGGGAACGATTGCATTAGGAGCTATTTCGTTTACCATGGATGCACTTCCGGG

TACACCGCAAATTCAAAATGTTATTCCAACGACTTTTTTAAAACAGACATCTATGCTGCTCCGACAC

TGGGGATTATCGGATCAGTTATTGTTTTTAGCTTAGGTCTTTGGTATTTAGAAAGCCGAAGTAAAAAA

GCAAGAAAAGCTGGAGAAGGCTACTACGGATTTAATAATGAACTAGCAGCTGCTGCAGAGCTAGAA

AAAGAGTCTGTACCTTCTACAGAACCTAAATATGAACCTAGCGTAGCAAGACAGATTTTAGCGTTTG

TTCCGCTCGTGCTTGTAGGGGTTGCAAACAAATTTTTACTGTTTCTATTCCAAAGTGGTACCCAGAC

GGTTTTGATTTTGCCAAAATTGGCCTTGAAGCGTTCGGAAAAGTAGATTTAACCGCAGTTGTTGGTAT

TTGGTCAGTGGAGTTAGCACTTGTTATTGGAATTATTACGACGATTGCGTATGACTGGAAGAGAGTG

ACCACCGGTTTCCAAGCGGGTCTTAACGCAAGTATTGGCGGAGCGCTTCTCGCTGCGATGAACACGG

GAGCTGAGTATGGCTTTGGAGGCGTTATTTCATCTCTTCCTGGCTTTGCAACCGTTCGCGATGGTATT

TCACATACCTTTACAAATCCGCTTGTCAATGGAGCCGTTACGACAAACATTTTAGCCGGTATTACAGG

ATCCGCTTCTGGAGGTATGGGAATTGCGCTTAGTGCGATGGGAGATAAGTATGTAGAAGCTATTAAT

CAATACAATATTCCTCCTGAAGTTATGCACAGAGTTGTAGCGATGGCATCAGGCGGTATGGATACGC

TTCCTCATAACGGAGCCGTTATTACCCTGCTTGCTATTACCGGGTTAACCCACAAGCAGTCTTACCGA

GATATTTTTGCCATCACTGTTATAAAAACGCTTGCATGTTTCTTAATCATTGGAATCTACAGCTTAACT

GGACTCGTATAA

Fig. S12. DNA sequence of *02676*

> ABDD91_RS04670 *actP*

ATGAACGTACTAGCTTTCTCACTTTTTTTAGCTATTGTAGGTCTTACGCTAATCATTACGTATGCGGCA

TCCAAGCGAACAAAAACGACCAGTGACTTTTATACAGCAGATGGAAGTTTAACCGGCTGGCAAAAC

GGCATGGCGATTGCCGGAGATTATATGTCTGCTGCGTCATTTTTAGGCATAGCTGGAATGGTAGCGCT

TTCTGGATTTGACGGTTTTTTTACAGCATCGGCTTTTTAGTTGCCTATCTCGTCGTGTTATACATTGT

AGCTGAACCACTTCGCAACTTAGGAAAGTACACGATGGCAGATATGATAGCAGCTCGTTTTAACGAA

AAGAAAGTGCGGGGAGTTGCCGCATTAAACACGATCACCATTTCCATTTTTTATATGATTGCTCAGCT

TGTTGGAGCAGGAGCATTAATTCATTTATTACTTGGTCTAGATTACGTGTATTCTGTCTTGATTGTCGG

CGTGTTGATGACGGTTTACGTTGTATTTGGAGGCATGACGGCGACCAGCTGGGTGCAGATTGTCAAA

GCCTTGCTTCTCATGATTGGTACGTTCATTATTTCCATTATTGTATTTTCAAAATTTGATTTTAGCTTTG

CTAAAATGTTTTCTGAGATGAAAACCGCAACTCCTCTTGGCGACGGATTTTTAAATCCAGGAAATAA

ATTCAAAAATCCGCTGGACATGATTTCGTTGAATTTAGCTCTTGTTCTTGGTACAGCAGGCCTTCCTC

ATATCCTTATTCGTTTCTTTACAGTAAAAGATGCCATCACCGCACGAAAGTCTGTTATTTATGCAACG

TGGATCATTGGCGTTTTTTACATTATGACAATCTTTTTAGGGTTCGGTGCTGCTGCATTCGTAGGGTAC

GATGATATTATCAAAGCAAATGCAGCAGGAAACATGGCAGCTCCCCTGCTCGCTCAAGTTCTTGGCG

GAGACTTTCTCTTTGCGTTTGTCTCAGCTGTAGCTTTTGCTACGATTTTAGCTGTTGTAGCTGGGCTGG

TGCTCTCAGCAGCTTCAGCATTCGCTCACGACTTTTATAGTCATATTCTCCGTAAAGGGGCTGCAACT

GAAAAAGAACAGGTAGTGGCTGCACGATGGGCTTCTATTGGGGTATCCATTCTATCTATTATTCTTGC

ATTATTTGCTCAAAACATGAACGTAGCATTTTTAGTTGCACTTGCATTTGCCGTAGCAGCGAGTGCTA

ACTTGCCAATTATTCTTCTTACTATTTTTTGGAAACGTTTTAATACAGCAGGAGCAGTCACTGGCATG

CTGGTTGGACTATTCAGCTCACTGTTTTTAGTTGCCATTAGTCCAAACGTGTGGGCACCTGAGCCTGG

AGCTGCTATCTTTGTAGGTGAGCCGCTCATCACATTAACAAACCCTGGTATTATCTCCATTCCTCTAG

GCTTCTTAGCTGCTTTTATAGGAACACTTCTTTCGTCTAAAAAAGCAGACGAAAAGAAATTTGATGA

GATTTTAGTAAAAGCAAATACAGGTATGGGAATCGAAGAGCCTGTCAAACATTAA

Fig. S13. DNA sequence of *actP*

> ABDD91_RS13400 *lutA_2*

ATGACGGCAACAAAAGACATTCAGCAAGCGTTTAAAGAACGGTTAGATTATGACGAGTTAATGAATT

GTATGAGATGCGGTTTTTGTTTGCCGAGCTGTCCGACGTATGGCCAAACCAATCAATATGAAGCAGC

GTCACCGCGCGGAAGAATTGCTTTGATGAAAGGAGTTGTAGATGGGCTTATTGAACCGGATGAATCG

GTTGAAAAGCAATTGAATCTTTGCCTTGGCTGCCGAGCGTGTGAGCCTGTTTGTCCTTCGGGTGTGAA

GTACGGACACTTATTAGAAGAAGCGCGTGATATTATTCAGCAGAAAAAACGACATAAATGGCCGGT

AAAAGCGCTCAGGCATATGGTGTTCGAACAGCTTTTTCCGCATAAAGAACGATTAAAAAATGTACAT

TCTCTGCTTGCTTTCTATCAAAGAAGCGGCTTGCAAAAAGCTGTTCAAAAAACAAATATATTAAACG

TCCTGCCCGGTAATTTAGCGCAAATGGAAAAAACGCTTCCGCCTGTGCCAACAAAAAAAGAAATGA

AGAAACGTCCTTTTTTATTTGAAGCAGAAGGAACAAGAGAGCGTACAGTTGCTTTTTTTACAGGGTG

TTTAATGGATACGATGTTTATGGAAACAAACAACGCGACGATTGCACTTTTACAAAAAGCGGGGTGT

AAGGTCGTTATACCAGAAGTGCAGACGTGCTGTGGCGCCCTGCATGCGCACGGCGGAGAAAAAGAT

CAGGCAAAAAAGTTAGCAAAACAAAATATTATGGCGTTTGAATCCATTCAAGCGGATGATATTGTCC

TTAATGCAGGCGGCTGCGGTGCGCTGTTAGTGGAATACGATCATCTATTAAAAGATGAGCCGGAATG

GAAAGAAAGAGCAGCGGTCTTTTCTGCAAAAGTCAAAGATTTCTCAGAGATTTTATTGCAGCAGCAG

TTTGTAGAAAGACAAAAGTTATCGCTTCCAAGTCAAATTATTACGTATCAAGATTCGTGTCATTTAAG

AAACGTGATGAAAACGTCCAGCGCACCGAGAAAACTGATCCAAGCGATTAACGGAACGGTGTTTAA

TGAAATGGAAAATGCGGATCATTGCTGCGGCTCAGCTGGTATTTATAACTTAACGGAACAAGAGATG

TCTATGCAAATTTTAGATTATAAAATGGAAAAAGTGAAAGAAGCCCATGCCCATACAATTGTAACAG

CAAACCCGGGCTGTCTCATTCAAATGAAGCTAGGCGTAGTCAGAGAAGGAGTAGAAGAAAGTGTAA

GGGCCGTTCATCTTGCAGATTTACTTCTTGAAGCAGTAGAAAGCAAATAA

Fig. S14. DNA sequence of *lutA_2*

> ABDD91_RS07970 *levE*

ATGAAAATCGTTTTAGCAAGAATTGATGACCGCTTTATTCACGGGCAGGTATTAACAAGATGGATCA

AAACAAATGCAGCAGATCGAATTATCATTGTTTCAGATGAGGTGGCAGCGGATGAAATGCGAAAAA

CGCTTATTCTTTCTGTGGCTCCTTCGAATGTAAAAGCAAGTGCTGTCTCAATTTCTAAAATGACAAAA

GCATTTCACAGCCCTCGTTATCAGGATACAACAGCCATGCTGCTGTTTGAAAGTCCTGCAGATATTGT

AGCTCTTGTTCAAGCTGGAGTGCCTATCGAGACAGTAAACGTAGGCGGCATGCGTTTTGCTAATGAC

CGAAAACAAATCACAAAATCTGTAAGCGTTACCGAAAAAGATATCGATGCTTTTGAGAAACTGCAG

GAACTAGGCGTAAAGCTTGAGCTGCGCCAATTACCCTCAGATTCTAGCGAAAATTTTATTCAACTACT

GAGAAATGAAACAAAAATCAAATAA

Fig. S15. DNA sequence of *levE*
